# A novel type of light-harvesting antenna protein of red algal origin in algae with secondary plastids

**DOI:** 10.1186/1471-2148-13-159

**Published:** 2013-07-30

**Authors:** Sabine Sturm, Johannes Engelken, Ansgar Gruber, Sascha Vugrinec, Peter G Kroth, Iwona Adamska, Johann Lavaud

**Affiliations:** 1 Ökophysiologie der Pflanzen, Fach 611, Universität Konstanz 78457 Konstanz, Germany; 2 Biochemie und Physiologie der Pflanzen, Fach 602, Universität Konstanz 78457 Konstanz, Germany; 3 Present address: Institute of Evolutionary Biology (CSIC-Universitat Pompeu Fabra), 08003 Barcelona,Spain; 4 Present address: Department of Biochemistry & Molecular Biology, Dalhousie University, Sir Charles Tupper Medical Building, 5850 College Street, Halifax, Nova Scotia B3H 4R2, Canada; 5 Present address: UMR 7266 CNRS-ULR ’LIENSs’, CNRS/University of La Rochelle, Institute for Coastal and Environmental Research, La Rochelle Cedex, France

**Keywords:** Complex plastids, Diatoms, Chloroplast, Gene transfer, Light-harvesting antenna proteins, Red lineage chlorophyll *a/b*-binding-like proteins

## Abstract

**Background:**

Light, the driving force of photosynthesis, can be harmful when present in excess; therefore, any light harvesting system requires photoprotection. Members of the extended light-harvesting complex (LHC) protein superfamily are involved in light harvesting as well as in photoprotection and are found in the red and green plant lineages, with a complex distribution pattern of subfamilies in the different algal lineages.

**Results:**

Here, we demonstrate that the recently discovered “red lineage chlorophyll *a/b*-binding-like proteins” (RedCAPs) form a monophyletic family within this protein superfamily. The occurrence of RedCAPs was found to be restricted to the red algal lineage, including red algae (with primary plastids) as well as cryptophytes, haptophytes and heterokontophytes (with secondary plastids of red algal origin). Expression of a full-length RedCAP:GFP fusion construct in the diatom *Phaeodactylum tricornutum* confirmed the predicted plastid localisation of RedCAPs. Furthermore, we observed that similarly to the fucoxanthin chlorophyll *a/c*-binding light-harvesting antenna proteins also RedCAP transcripts in diatoms were regulated in a diurnal way at standard light conditions and strongly repressed at high light intensities.

**Conclusions:**

The absence of RedCAPs from the green lineage implies that RedCAPs evolved in the red lineage after separation from the the green lineage. During the evolution of secondary plastids, RedCAP genes therefore must have been transferred from the nucleus of the endocytobiotic alga to the nucleus of the host cell, a process that involved complementation with pre-sequences allowing import of the gene product into the secondary plastid bound by four membranes. Based on light-dependent transcription and on localisation data, we propose that RedCAPs might participate in the light (intensity and quality)-dependent structural or functional reorganisation of the light-harvesting antennae of the photosystems upon dark to light shifts as regularly experienced by diatoms in nature. Remarkably, in plastids of the red lineage as well as in green lineage plastids, the phycobilisome based cyanobacterial light harvesting system has been replaced by light harvesting systems that are based on members of the extended LHC protein superfamily, either for one of the photosystems (PS I of red algae) or for both (diatoms). In their proposed function, the RedCAP protein family may thus have played a role in the evolutionary structural remodelling of light-harvesting antennae in the red lineage.

## Background

Higher plants, algae and cyanobacteria absorb light energy to drive oxygenic photosynthesis. Light harvesting is the first step in the photosynthetic process and is mediated by pigment-binding proteins forming light-harvesting antenna systems. However, excess light can be harmful and can lead to protein damage due to the formation of reactive oxygen species (ROS), establishing a strong evolutionary pressure on photosynthetic organisms to develop potent photoprotective mechanisms [1-3]. Both functions, light harvesting and photoacclimation/photoprotection are mediated by members of the extended light-harvesting complex (LHC) protein superfamily in photosynthetic eukaryotes [1,3-9]. The eukaryotic members of the extended LHC protein superfamily have a common origin and evolved from a cyanobacterial one-helix ancestor with a characteristic chlorophyll-binding motif that is strongly conserved across the entire extended LHC protein super family [1,4-6,8,10,11]. Apart from LHC superfamily proteins also other proteins are known to bind chlorophyll, examples are the prochlorophyte Chl *a/b* binding proteins [12] or the IsiA chlorophyll-binding protein in cyanobacteria [13]. The chlorophyll binding motifs of these proteins are non-homologous to motifs found in the LHC protein super family [5,12].

Eukaryotic photosynthetic organisms evolved by the uptake of an ancient cyanobacterium and the subsequent reduction of the endosymbiont to an organelle. Soon after the evolution of primary plastids, photosynthetic eukaryotes split into three lineages, chlorophytes (green algae and land plants), rhodophytes (red algae) and glaucophytes [14,15]. During this process, structure and composition of the light-harvesting systems changed: Phycobilisomes, the main light harvesting systems in cyanobacteria, were lost in chlorophytes and their function was taken over by members of the extended LHC protein superfamily. Rhodophytes and glaucophytes, however, retained phycobilisomes as a part of their light-harvesting machineries [5,15].

Diatoms and cryptophytes (along with related algal groups collectively termed “Chromista”) evolved via secondary endocytobiosis, the uptake of a eukaryotic alga into a eukaryotic host cell [14-16], with the secondary endosymbiont being phylogenetically related to recent red algae [17]. Red algae and algae with secondary plastids of red algal origin are therefore often collectively referred to as the “red lineage” of photosynthetic eukaryotes, opposed to the “green lineage” (chlorophytes and organisms with secondary plastids of chlorophyte origin).

Interestingly, also secondary endocytobiosis led to drastic changes in structure and function of the light-harvesting systems in the red lineage. In cryptophytes, phycobilins are present, however they are not organised in phycobilisomes, while diatoms exclusively use LHC superfamily proteins for light harvesting [5,15].

Across all recent bacterial and eukaryotic photosynthetic organisms, the extended LHC protein superfamily consists of the LHC, LHC-like and PSBS protein families. The LHC protein family in the red lineage is represented by LHCR proteins present in red algae (“R” for Rhodophyta), chlorophyll (Chl) *a/c*-binding (CAC) proteins present in algal groups with secondary plastids of red algal origin, also called fucoxanthin CAC proteins (FCPs) or LHCF (“F” for fucoxanthin) in diatoms and brown algae, LI818, called also LHCX in diatoms, and a less known clade, LHCZ, described for some algae with complex plastids [4-6,8,9,18]. In the green lineage, the LHC protein family is represented by Chl *a/b*-binding (CAB) proteins and LI818, also called LHCSR in green algae [4-6,9,19].

The LHC-like protein family is divided into early light-induced proteins (ELIPs), stress-enhanced proteins (SEPs, also called light-harvesting-like (LIL) proteins), one-helix proteins (OHPs, also called high light-induced proteins HLIPs), and high light (HL) intensity-inducible LHC-like 4 (LHL4) proteins [1,20]. While ELIPs and LHL4 are found exclusively in the green lineage, SEPs and OHPs are shared between red and green algae [8,11,20]. Two types of OHPs can be distinguished: the OHP1/HLIP-type present in cyanophages, cyanobacteria and photosynthetic eukaryotes and the OHP2-type restricted to eukaryotic organisms [8,11]. Members of the PSBS protein family are present only in the green lineage [5,11].

Proteins from the CAC, LHCR and CAB protein families mainly fulfill a light harvesting function, while members of the LHC-like, LI818/LHCX/LHCSR, PSBS and LHL4 families are mainly involved in photoprotection and photoacclimation. It was proposed that these proteins play a role in the regulation of Chl and tocopherol biosynthesis, participate in the transient binding of released free Chlorophylls, thus preventing the formation of ROS, and act as a sink for excessive excitation energy in a process called non-photochemical quenching (NPQ) [9,20].

Four novel sequences belonging to the extended LHC protein superfamily were recently reported from the red algae *Galdieria sulphuraria* and *Griffithsia japonica* and from the two diatoms *Phaeodactylum tricornutum* and *Thalassiosira pseudonana*[11]. Based on sequence similarity (hidden Markov model analysis and BLAST searches) and predicted secondary structure (presence of three predicted transmembrane *α*-helices) these sequences did not fall into any of the previously described extended LHC protein superfamily groups but formed a new group instead, termed red lineage CAB-like proteins (RedCAPs) [11]. Here, we elucidate the taxonomic distribution, phylogeny, localisation, expression and potential function of these not yet characterised RedCAPs.

## Results and discussion

### Taxonomic distribution of RedCAPs

To investigate the taxonomic distribution of RedCAP sequences, we searched publicly available expressed sequence tag (EST) and genomic databases and found orthologs in Cryptophyta, Haptophyta, Heterokontophyta (e.g. diatoms, brown algae and others) and Rhodophyta. No RedCAP sequences were found in organisms of the green lineage of photosynthetic eukaryotes, while genomes from organisms of the red lineage of photosynthetic eukaryotes were generally found to encode RedCAPs (Table 1, Table S1, see Additional file 1). Thus, the presence of RedCAPs is restricted to red algae and photosynthetic Chromista with secondary plastids of red algal origin (Table 1).

**Table 1:** Taxonomic distribution of RedCAP sequences in red algae with primary plastids and algae with secondary plastids of red algal origin

**Classification (class, species)**	**Database [references]**	**Gene model**
Cryptophyceae		
*Guillardia theta*	JGI [65,66]	Guith1:186670
Coccolithophyceae		
*Emiliania huxleyi*	JGI [68]	Emihu1:310333
*Isochrysis galbana*	GenBank EST [70]	gi106825476
Pavlovophyceae		
*Diacronema lutheri*	GenBank EST [70]	gi106858477
Phaeophyceae		
*Ectocarpus siliculosus*	GenBank EST [21,70]	gi242173528
	OrcAE [22,23]	Esi0256_0036
Bacillariophyceae		
*Fragilariopsis cylindrus*	JGI [68]	Fracy1:210193
*Phaeodactylum tricornutum*	JGI [61,62]	Phatr2:17326
Coscinodiscophyceaes		
*Thalassiosira pseudonana*	JGI [63,64]	Thaps3:270215
Dictyochophyceae		
*Pseudochattonella farcimen*	GenBank EST [24,70]	gi319967268
Pelagophyceae		
*Aureococcus anophagefferens*	JGI [25,68]	Auran1:25646
Florideophyceae		
*Furcellaria lumbricalis*	GenBank EST [26,70]	gi294363890
*Gracilaria changii*	GenBank EST [70]	gi120457728
*Gracilaria tenuistipitata*	GenBank EST [70]	gi327362708
*Griffithsia japonica*	UniProt [75]	Q7XZ09
*Griffithsia okiensis*	GenBank EST [27,70]	gi224829379
Cyanidiophyceae		
*Galdieria sulphuraria*	Michigan State University	contig 9803
	*Galdieria* database[72-74]	
Bangiophyceae		
*Pyropia yezoensis*	GenBank EST [28,70]	gi8590586
Porphyridiophyceae		
*Porphyridium purpureum*	GenBank EST [29,70]	gi317790494

Interestingly, in contrast to members of the LHC and LHC-like families, but similar to the PSBS family, almost all identified RedCAPs are encoded by single-copy genes. The only possible exceptions are the haptophyte *Emiliania huxleyi* which possesses an additional, possibly degenerated RedCAP sequence (Table S1, see Additional file 1) and the red alga *Cyanidioschyzon merolae*, which apparently does not possess a RedCAP gene, possibly due to its overall highly reduced genome [30].

### Unique phylogenetic position of RedCAPs within the extended LHC protein superfamily

First we analysed RedCAP sequences in order to resolve their phylogenetic position among the three- and four-helix protein families of the extended LHC protein superfamily. We used the conserved Chl-binding motif present in helices I and III and obtained a sequence alignment that consists of 51 amino acid positions (26 and 25 amino acid positions from helices I and III, respectively (see Additional file 2)), 45 out of the 51 amino acid positions are not fixed and polymorph in more than one taxon and hereby contribute to the phylogenetic information in the analysis.

Alignments of RedCAP amino acid sequences with three-helix members from the extended LHC protein superfamily present in the red (FCP/LHCF, LHCR or LHCX) and green (CAB, LHCSR, ELIP and LHL4) algal lineages confirmed their distinct primary and secondary structure (Figure S1 (A) and (B), see Additional file 3). In all three-helix members of the LHC protein superfamily investigated so far only helices I and III are conserved while helix II shows much lower sequence conservation; in contrast to this, sequence conservation also occurred in the second helix of RedCAPs from different organisms (Figure S1 (C), see Additional file 3). This conserved region also included residues that might be involved in pigment binding (Figure S2, see Additional file 4). Pigment binding depends on the three dimensional folding of the actual protein (which again depends on the presence of pigments) and the protein/lipid surrounding of the folded protein; so it is difficult to predict. Overall, there were fewer potential pigment binding sites in RedCAPs than in LHCs, which might indicate that RedCAPs are possibly less chlorophyll loaded than LHCs and possibly fulfill a different function.

The alignment was also used to build a phylogenetic tree (Figure S3, see Additional file 5), the RedCAP sequences clearly clustered together and formed a well-defined, monophyletic clade within the extended LHC protein superfamily. This pattern was also observed in an analysis of the extended LHC protein superfamily based on the first Chl-binding helix including one- and two-helix LHC-like proteins (not shown). Therefore, not only the differences in the second transmembrane helix, but also the ones in helices I and III placed RedCAPs into a clade distinct to that of LHCs.

**Figure 1:**
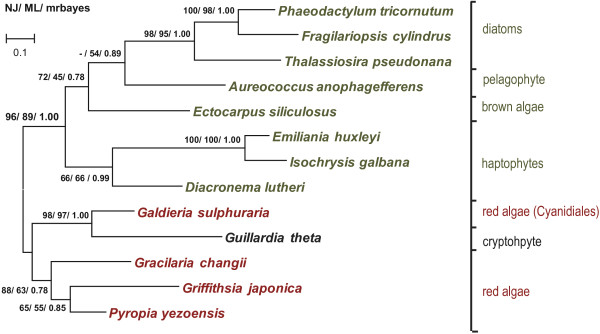
**Phylogenetic relationships of RedCAPs.** Thirteen available full-length sequences from red algae and algae with secondary plastids of red algal origin, with an aligned 146 amino acid positions, thereof 99 phylogenetically informative, were analysed. A Maximum likelihood tree was inferred under the CpRev + G model. Bootstrap values for Neighbor-joining (10,000 replicates) and Maximum likelihood analysis (100 replicates) as well as posterior probabilities (one million generation, 25% burn-in) are given. Accession numbers of analysed sequences are listed in Table S1, see Additional file 1, for sequence alignment see Additional file 6.

In a phylogenetic analysis of all currently available RedCAP sequences (RedCAPs from 13 taxa, alignment of 146 positions, see Additional file 6), the expected species tree with red algae and algae with complex plastids as sister groups was recovered to some detail (Figure 1). This implies that the evolution of RedCAPs apparently did not involve the emergence of paralogous gene copies (as it is commonly observed in LHCs) and that no horizontal gene transfer events could be detected within algae with secondary plastids of the red lineage.

Based on primary sequence similarities, conservation patterns (Figure S1, see Additional file 3) and phylogenetic analyses (Figure S3, see Additional file 5), RedCAPs formed a distinct family within the extended LHC protein superfamily that was neither more closely related to other three-helix families, like LHC, LHC-like or LHL4, nor to the four-helix PSBS family.

### Complex evolutionary history of RedCAP genes

**Figure 2:**
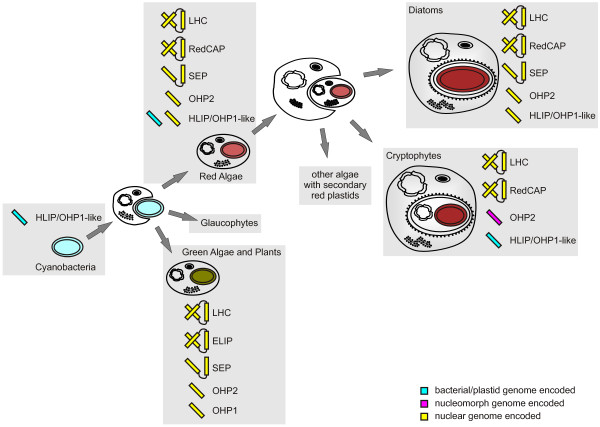
**Proposed evolutionary history of RedCAP, LHC and LHC-like genes.** RedCAPs evolved in red algae after primary endocytobiosis and their genes can be found in the nuclear genome of almost all red algae investigated so far. During secondary endocytobiosis, when a red alga was taken up by another eukaryotic host, the RedCAP gene was transferred to the nucleus of the host cell and was lost from the nuclear genome of the former endosymbiont. Similarly, different nuclear encoded LHC variants evolved in organisms with primary plastids and were transferred to the nucleus of the secondary host during the evolution of secondary plastids. Interestingly, in diatoms, HLIP/OHP1-like genes (plastid encoded in red algae) as well as OHP2 genes (nucleus encoded in red algae) have been transferred to the nucleus of the secondary host cell. This is in contrast to the situation in the cryptophyte *Guillardia theta*, in which HLIP/OHP1-like is plastid encoded, and OHP2 is nucleomorph encoded. Table S2 (see Additional file 7) contains detailed genome/gene information.

Evolutionary studies indicate that all LHC superfamily members have a common origin and arose from an cyanobacterial one-helix HLIP-like protein ancestor [4-6,8-11,15]. The HLIP/OHP1-like sequences were likely at the origin of the nuclear-encoded OHP1, OHP2 and SEP in the green or OHP2 and SEP in the red algal lineage (the monophyletic group of OHP2 sequences might also originate from degenerated SEP sequences) [11]. In the green lineage, the ancestral HLIP/OHP1-like sequences were lost, whereas in the red lineage HLIP/OHP1-like sequences can be found, encoded either on the plastid or the nuclear genomes (Figure 2, Table S2, see Additional file 7).

Remarkably, all LHC protein superfamily members with more than one transmembrane helix containing chlorophyll binding motifs were exclusively found in eukaryotic photosynthetic organisms and are encoded on the nuclear genomes, which implies that they evolved after the endosymbiotic gene transfer from the cyanobacterial genome to the nuclear genomes of the ancestors of chlorophytes, rhodophytes and glaucophytes (it is noteworthy that a fusion protein with two predicted TM helices was reported in a strain of the cyanobacterium *Synechococcus*[31], this protein is not related to the two helix SEPs [11] and so far has not been found in any eukaryote). The ancestor of LHCR/CAB or ELIPs and RedCAPs in green or red algae, respectively, evolved by independent, internal gene duplication, likely from different SEP groups after the initial gene transfer [11].

After secondary endocytobiosis, LHCR genes were transferred to the nucleus of the secondary host cell and gave rise to LHCF and CAC genes in Heterokontophyta and Cryptophyta, respectively [18]. Similarly, RedCAP and SEP genes were transferred to the host nucleus in Heterokontophyta and Cryptophyta (Figure 2, Table S2, see Additional file 7). Comparing the location of HLIP/OHP1-like and OHP2 genes a striking difference between Heterokontophyta and Cryptophyta became apparent; while in Heterokontophyta, HLIP/OHP1-like and OHP2 genes have been transferred to the nuclear genomes and subsequently got lost from the secondary endosymbiont genome (which does not persist any more), HLIP/OHP1-like and OHP2 sequences were not transferred to the nucleus in cryptophytes, instead they can still be found on the plastid or nucleomorph genomes, respectively (with the notable exception of the non-photosynthetic *Cryptomonas paramecium* which lost the plastid and nucleomorph encoded HLIP/OHP1-like and OHP2 genes) (Figure 2, Table S2, see Additional file 7).

### RedCAPs are targeted to the complex plastids of diatoms

**Figure 3:**
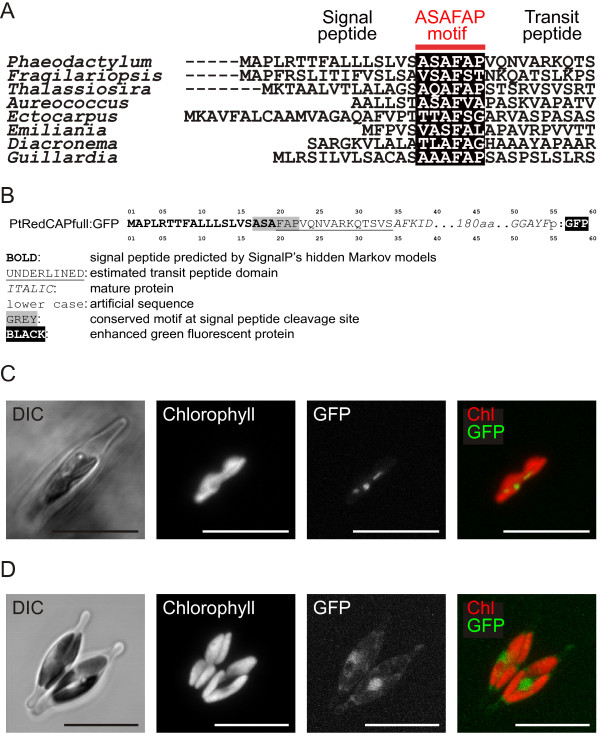
**Localisation of the RedCAP protein in complex plastids of diatoms.(A)** Bipartite plastid targeting sequences in photosynthetic Chromista. The conserved “ASAFAP”-motif [32,33] at the interface between signal and transit peptides is marked. **(B)** Sequence of the *Phaeodactylum tricornutum* RedCAP full length GFP fusion construct. **(C)** Expression of the full-length RedCAP:GFP fusion constructs in *P. tricornutum*. **(D)** Expression of GFP without targeting pre-sequence in *P. tricornutum*. Panels show microscopical images of transmitted light (differential interference contrast, DIC), chlorophyll autofluorescence, GFP fluorescence and a merged image from left to right, fluorescence images are maximum intensity projections of 14 slices of a 4.9 *μ*m image stack, scale bars represent 10 *μ*m.

All identified RedCAPs in algae with secondary plastids were nuclear-encoded and include an N-terminal bipartite pre-sequence, consisting of a signal and a transit peptide domain and a conserved “ASAFAP”-motif located at the interface between both domains, which is required for import through the four membranes surrounding such plastids [32,33]. This suggests a plastid location of RedCAPs (Figure 3A). To verify the predicted location experimentally, we fused the full-length RedCAP sequence to the green fluorescent protein (GFP) gene (Figure 3B) and expressed it in the diatom *Phaeodactylum tricornutum*. Analysis of the GFP signal by confocal fluorescence microscopy revealed that this signal co-localised with the red Chl autofluorescence (Figure 3C, Figure S4, see Additional file 8), thus confirming a plastid localisation of RedCAP in diatoms.

Similarly, also the nucleus encoded OHP2 of *P. tricornutum* was reported to be targeted to the plastid [33] (the construct name in the cited study is PtHlip2:GFP).

This shows that gene transfers from the nucleus of the secondary endosymbiont to the nucleus of the secondary host cell were accompanied by acquisition of targeting pre-sequences that are suitable to re-target the gene product to its original location. This process is not trivial, since plastid targeting pre-sequences of red algae show completely different features than plastid targeting pre-sequences of diatoms [34], and pre-sequence acquisition is considered to be a crucial step in the evolutionary reduction of organellar genomes [35].

### RedCAPs show a unique expression pattern under light stress conditions

**Figure 4:**
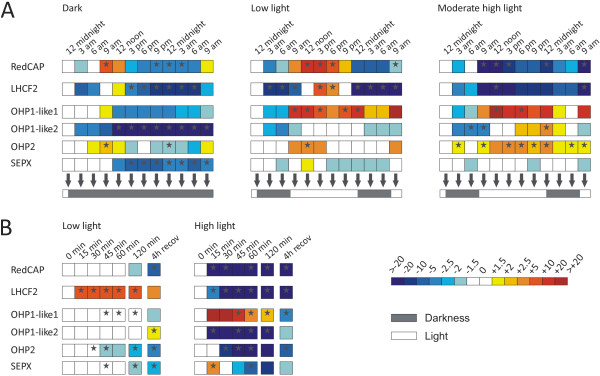
**Expression of RedCAP and selected members from LHC and LHC-like protein families in diatoms. (A)** Cells of *P. tricornutum* preadapted to LL (45 *μmol photons*·*m*^−2^·*s*^−1^) with illumination from 8 am to 12 midnight were either kept at LL conditions or transferred to D (no illumination) or ML (750 *μmol photons* ·*m*^−2^·*s*^−1^ throughout the illumination period) for 33 h and samples were collected every 3 h. Dark and light periods are indicated by grey or white bars, respectively, at the bottom of the expression data. **(B)** Cells of *P. tricornutum* preadapted to LL for 6 h (the first 6 h of the regular illumination period) were either kept at LL for additional 6 h or exposed to HL (1,500 to 2,000 *μmol photons* ·*m*^−2^·*s*^−1^) for 2 h and transferred back to LL for recovery (recov) for 4 h, samples were taken at the times indicated (relative to the transfer into HL). Relative transcript levels were calculated with help of the Relative Expression Software Tool REST [102] using the first sample of each light condition as a calibrator and 18 S rDNA as an endogenous control. The colour code indicates relative gene expression values as indicated by the scale bar on the right. Shown expression levels are average from four independent experiments, grey stars in the coloured boxes mark significant changes compared to the first sample as indicated by the statistical randomisation tests by REST [102].

We investigated the expression of the *P. tricornutum* RedCAP gene and compared it to the expression of selected members of the LHC and LHC-like families. Cells were pre-adapted to low light (LL) at 16 h of daily illumination. With the onset of the dark period, cells were either kept in the same condition (LL) or transferred to continuous darkness (D) or moderate hight light (ML) for one regular 16 h illumination period. Transcript levels of selected genes were assayed in 3 h intervals throughout the following 33 h (Figure 4 and Table S3, see Additional file 9). In the LL condition (the regular culture condition), LHCF2 transcript levels were significantly down-regulated in the dark period and significantly up-regulated in the light period compared to the transcript level at the onset of darkness. This is consistent with previous reports of light dependent diurnal transcript regulation for this gene [36]. Following a similar pattern, also transcript levels of RedCAP and OHP1-like 1 were significantly up-regulated during the light period and down-regulated (no significant difference compared to the transcript level at the onset of darkness) during darkness. A similar expression pattern of RedCAP upon a shift from D to LL was recently reported [37]. In the D condition (no illumination) the amounts of LHCF2 and RedCAP transcripts were significantly down-regulated, although a transient up-regulation of the transcript level was measured at the time when the light was previously switched on, this effect was also observed (however, not statistically significant) for LHCF2 (Figure 4 and Table S3, see Additional file 9). In the ML condition (illumination with moderate hight light throughout the 16 h light period), RedCAP and LHCF2 transcripts were down-regulated compared to the transcript level at the onset of darkness, independent of the light or dark phase (Figure 4A, Figure S5 see Additional file 10). Thus, we can conclude that RedCAP and LHCF2 show a diurnal regulation of the gene expression at LL, which is not maintained in D or under ML illumination. This is in agreement with previous studies showing diurnal regulation of LHCF2 genes [36,38] and with the clustering of the *P. tricornutum* RedCAP gene with LHCF and LHCF-like genes in a hierarchical clustering analysis of diatom ESTs obtained from a range of different environmental conditions [39].

LHCFs were reported to be transcriptionally repressed in response to high light (HL) in the diatom *P. tricornutum*[40], while LHCX are induced by HL in *P. tricornutum*[40,41] and other diatoms [38,42,43] confirming their role in photoprotection [44]. The OHPs and SEPs were shown to accumulate in response to HL in *Arabidopsis thaliana*[20] but nothing is known about their expression and function in diatoms. To investigate whether RedCAP is induced in response to HL, we exposed LL-pre-adapted *P. tricornutum* cells to HL for 2 h and transferred them back to LL for recovery for additional 4 h. The control cultures were kept at LL for the same amount of time. The transcript levels of RedCAP, LHCF2, OHP1-like 2, OHP2 and SEPX were significantly down-regulated after the onset of HL illumination (Figure 4B, and Table S3, see Additional file 9). The observed expression patterns of LHCF2 and RedCAP during a LL to HL shift were similar to the ones reported by Nymark et al. [37,40]. The transcript level change of LHCF2 during a shift from LL to HL also seems to be reflected by a change of its protein amount [45]. For RedCAP and LHCF2, after 4 h of recovery the transcript level remained low, while for OHP1-like 2 and OHP2, the transcript level after 4 h of recovery was closer to the initial transcript level. In contrast, the transcript level for OHP1-like 1 was transiently up-regulated during the first 30 min of HL exposure and decreased below the level present in LL-kept culture during recovery (Figure 4B). The different expression patterns of OHP1-like 2, OHP2 and SEPX genes as compared to *A. thaliana* orthologs [20] suggest that in diatoms, these proteins might perform other functions than photoprotection. A similar down-regulation of HLIP/OHP1 and OHP2 transcripts in response to HL was also reported for the Cryptophyte *Guillardia theta*[46].

It was recently demonstrated that RedCAP is associated to photosystem I (PS I), together with LHCR and some LHCF, both in centric and in pennate diatoms [47,48]. Other studies using different isolation procedures [45,49] have reported RedCAPs in the “whole pool” of LHCF complexes which is shared between PS I and photosystem II (PS II) [45]. These findings might be explained by either 1) loose binding of RedCAP at the periphery of PS I, although no exact location has been proposed by the recent mapping of the diatom PS I [48], or by 2) loose binding of RedCAP to the periphery of LHCF complexes that are associated to both photosystems. Considering the diurnal and light dependent regulation of the RedCAP transcript levels (our results and [37]), RedCAP appears not to be light stress-induced and therefore is obviously not involved in HL photoprotection. Instead, RedCAP is a fast responding gene during a shift from prolonged D to LL [37] but not during a shift from HL to LL (this study). RedCAP was also found under low blue and red light conditions in amounts similar to other LHC related proteins (LHCF, LHCR and LHCX) [50]. Interestingly, LHCF2, which shows a similar light-dependent expression as RedCAP, was recently proposed to be more closely associated with PS I than with PS II and to bind fucoxanthin pigments that change the light absorption properties of the LHCF antenna (i.e. absorption is shifted towards blue wavelengths) [45]. We therefore hypothesise that RedCAPs might be involved in the light-dependent structural and/or functional reorganisation of the light-harvesting antenna of PS I and/or PS II in response to D/LL shifts (including water induced changes in light quality) as diatoms regularly experience in their natural habitats, i.e. the water column, mudflats or sea-ice packs [51-53]. The recent report by Nymark et al. [37] shows a deep light-dependent reorganisation of the diatom photosynthetic apparatus during dark-light shift conditions. This also includes components of the carbon metabolism [54] and requires a fine tuning between light (intensity and quality), the photosynthetic activity and gene regulations [41,55,56].

## Conclusions

Based on the expression pattern of RedCAP transcripts that resembles that of LHCF2 and differs from LHC-like family members as well as based on their localisation in the thylakoid membrane with an association at the periphery of LHCF complexes associated to PS I and PS II [45,47-49], we propose that RedCAPs act as antenna-associated proteins in diatoms and related algae. There are striking differences in the regulation of photosynthesis between plants and diatoms [51,57], especially in the structural organisation of the light harvesting antenna systems [58,59]. The orphan phylogenetic position of RedCAPs together with an expression pattern similar to LHCF2 transcripts promote this group as an interesting candidate to explain these differences.

Major shifts in the functional organisation of the different light harvesting systems occured in early algal evolution [8,9]. Remarkably, in plants as well as in diatoms, phycobilisomes as antenna proteins have been convergently evolutionary replaced by members of the extended LHC protein superfamily. Accompanying this process, in green algae and plants, the PSBS and LHC-like protein families increased the diversity of subfamilies within the extended LHC protein superfamily [11]. Our study shows, that also in red algae and algae with secondary plastids of red algal origin the extended LHC protein superfamily diversified, among others, by the evolution of the RedCAP family.

Considering the global significance of diatoms and other algae with secondary plastids of the red lineage in the contemporary oceans [52] and the extraordinary photosynthetic efficiency and the high productivity of these organisms [51-53,60], elucidating the exact functional role of RedCAPs constitutes an important task for future studies.

## Methods

### Sequence search and annotation

The *Phaeodactylum tricornutum* v2.0 [61,62], *Thalassiosira pseudonana* v3.0 [63,64] and *Guillardia theta* CCMP2712 v1.0 [65,66] genome databases were accessed online via the United States Department of Energy Joint Genome Institute (JGI) genome portal [67,68] using TBLASTN and BLASTP [69]. Additional sequence data were collected from public databases including the National Center for Biotechnology Information (NCBI) [70] databases, the *Cyanidioschyzon merolae* Genome Project [30,71] database, the Michigan State University *Galdieria* Database [72-74], UniProt [75] and JGI [68]. Special attention was given to the nucleomorph genomes of the cryptophytes *Guillardia theta*[76], *Cryptomonas paramecium*[77], *Hemiselmis andersenii*[78] and *Chroomonas mesostigmatica*[79] as well as to the plastid genomes of the red algae *Cyanidioschyzon merolae*[80], *Cyanidium caldarium*[81], *Porphyra purpurea*[82], *Pyropia yezoensis*[70] and *Gracilaria tenuistipitata* var. *liui*[83], the cryptophytes *G. theta*[84], *Rhodomonas salina*[85] and *Cryptomonas paramecium*[86] and of the diatoms *P. tricornutum* and *T. pseudonana*[87]. The newly identified sequences of the extended LHC protein superfamily were classified according to their predicted secondary structures as well as sequence similarity to known Chl-binding proteins as described by Engelken et al. [11]. It should be noted that the *P. tricornutum* RedCAP is also known as “LHL1” in recent publications [37,45,47]. In this nomenclature, “LHL” stands for “light-harvesting-like”, a term that is ambiguous with the abbreviation “LIL” (also for “light-harvesting-like” [8]), which is why we prefer to use the name “RedCAP”. Prediction of transmembrane helices was done with the DAS algorithm [88], which is optimised for prokaryotic membrane proteins and therefore is well suited for proteins targeted to plastid membranes. Signal peptides were identified using the program SignalP 3.0 [89,90]. Bipartite chloroplast targeting pre-sequences were manually predicted by their characteristic N-terminal sequence motif [32,33].

### Phylogenetic analysis

Alignments were prepared with T-Coffee [91,92] and manually refined in BioEdit [93]. Due to the scarcity of gaps and insertions within and nearby the analysed transmembrane helices, Chl-binding sequence motifs can be easily aligned. Due to the partially high sequence divergence between the different LHC subfamilies, we restricted the analysis to a conservative alignment containing a combined stretch of 51 amino acid positions. With 45 parsimony informative sites, these amino acid positions were highly informative. Bootstrap values (10,000 replicates) for the Neighbor-joining analyses were obtained in MEGA5 [94]. Maximum likelihood bootstrap analyses with 100 replicates were performed using MEGA5 and PhyML [95,96] and posterior probabilities were calculated using MrBayes (2 million generations, 50% burn-in) [97], using a WAG+ *Γ*4 model of amino acid evolution. The amino acid substitution model was chosen using best maximum-likelihood fits as implemented in MEGA5 [94].

### Localisation studies

The RedCAP:green fluorescent protein (GFP) was constructed via standard cloning procedures [98] using the *P. tricornutum* transformation vector pPha-T1 (GenBank AF219942) [99] following strategies described earlier [32,33]. The RedCAP:GFP construct was sequenced (GATC Biotech AG, Konstanz, Germany) to ensure correct cloning. Nuclear transformation of *P. tricornutum* was performed according to the procedure described by Kroth [100]. Transformed cell lines were screened for the expression of GFP using an Olympus BX51 epifluorescence microscope (Olympus Europe, Hamburg, Germany). Images were then acquired with a confocal laser scanning microscope LSM 510 META (Carl Zeiss MicroImaging GmbH, Göttingen, Germany) using a Plan-Apochromat 63x/1.4 Oil DIC objective. For the images presented in Figure 3, GFP fluorescence and chlorophyll autofluorescence were excited at 488 nm, filtered with a beam splitter (HFT 405/488/543), and detected by two different photomultipliers with a band-pass filter (BP 505-530) for GFP fluorescence and a low pass filter (LP 650) for chlorophyll autofluorescence, transmitted light images were simultaneously detected. For the images presented in Figure S4 (see Additional file 8), GFP and Chl fluorescence were excited at 488 nm and detected simultaneously by the meta detector with spectral resolution (lambda mode) at 16 bit dynamic range and later separated via linear unmixing using the software ZEN (Carl Zeiss MicroImaging GmbH, Göttingen, Germany). Reference spectra were beforehand acquired from wild type cells (for Chl autofluorescence) and a transformed cell line expressing cytosolic GFP with spatial separation from the plastidic Chl autofluorescence (for GFP fluorescence). Transmitted light images (488 nm wavelength) were recorded separately after the fluorescence image stacks were completed. For both sets of images, maximum intensity z-projections were calculated from slices of image stacks to ensure complete detection of fluorophores within a cell.

### Diatom cultivation and light treatments

*Phaeodactylum tricornutum* (UTEX Collection, strain 646) was cultured in f/2 seawater medium [101] prepared with “Tropic Marin” artificial seawater (Dr. Biener GmbH, Wartenberg/Angersbach, Germany) at a final concentration of 50% (w/v) compared to natural seawater and continuously bubbled with sterile air. Cells were grown at 22°C at low light (LL, 45 *μmol*·*photons*·*m*^−2^·*s*^−1^) under a light regime of 16 h light/8 h dark (illumination from 8 am to 12 midnight). For the diurnal rhythm experiments, cultures were grown at LL for 4 days and cells at mid-logarithmic growth phase were kept further at LL or transferred to ML conditions (750 *μmolphotons*·*m*^−2^·*s*^−1^ throughout the illumination time) or D (no illumination) for 33 h. For the light stress experiments, cells grown at LL as described above. Starting at 2 pm (after 6 h of illumination) the cultures were either kept at LL for an additional 6 h or transferred to HL (1,500 to 2,000 *μmolphotons*·*m*^−2^·*s*^−1^) for 2 h and moved back to LL for 4 h for recovery. Photosynthetic active radiation was measured using a quantum photometer (Model LI-185A, Li-Cor Inc., Lincoln, NE, USA). Cells were harvested by centrifugation at 3,000 g for 1 min at 21°C and pellets were stored at -80°C prior to analysis. Four independent experiments were performed for each data point.

### Expression studies

The harvested cells were mechanically disrupted with mortar and pestle under liquid nitrogen and total RNA was isolated using a combination of phenol/chloroform extraction with Trizol reagent (Invitrogen, Carlsbad, CA, USA) and the RNeasy kit (Qiagen, Hilden, Germany). Genomic DNA contaminations were removed using Turbo DNase (Ambion, Woodward, TX, USA) according to the manufacturer’s instructions. 350 ng DNA-free RNA was reverse transcribed for each sample individually with the QuantiTect reverse transcription kit (Qiagen, Hilden, Germany). The resulting cDNA preparations were diluted 4-fold in RNase/DNase-free water and 1 *μ*L of the cDNA template was used in a 20 *μ*L qPCR reaction containing PCR primers (primer sequences in Table S4, see Additional file 11) and DNA polymerase master mix with SYBR Green (MESA GREEN qPCR MasterMix Plus for SYBR Assay Low ROX, Eurogentec Deutschland GmbH, Cologne, Germany). The reaction was heated to 95°C followed by 40 cycles for 15 s at 95°C and 1 min at 60°C. The amount of amplified DNA was monitored by measuring fluorescence at the end of each cycle using the Real-Time PCR System 7500 (Applied Biosystems, Lincoln, CA, USA). Relative transcript levels were calculated with help of the Relative Expression Software Tool REST [102] using the first sample of each light condition as calibrator and 18 S rRNA as endogenous control. 18 S rRNA (GenBank: AY485459.1) has been identified as one of the most stable endogenous controls for qPCR in *P. tricornutum*[41,103]. The *P. tricornutum* LHCF2 gene is a gene for which light/dark dependent up/down regulation has been demonstrated previously [36] (LHCF2 is called “FcpB” in the cited study). We therefore included LHCF2 as a positive control for transcript up- and down-regulation using the exact primer sequences proposed by Siaut et al. [36]). Gene models of the investigated sequences from *P. tricornutum* can be accessed at the JGI *P. tricornutum* v2.0 genome database [61,62] with the following protein IDs (in parentheses): LHCF2 (25172), OHP1-like 1 (53712), OHP1-like 2 (33932), OHP2 (55112), SEPX (56446) and RedCAP (17326).

## Abbreviations

CAB: Chlorophyll a/b-binding; CAC: Chlorophyll a/c-binding; Chl: Chlorophyll; D: Darkness; ELIP: Early light-induced protein; GFP: Green fluorescent protein; HL: High light; HLIP: High light-induced protein; LHC: Light-harvesting complex; LHCF: Fucoxanthin-containing light-harvesting complex; LHCR: Light-harvesting complex in red algae; LHL: High light intensity-inducible light-harvesting complex-like; LL: Low light; ML: Moderate high light; NPQ: Non-photochemical quenching; OHP: One-helix protein; PS I: Photosystem I; PS II: Photosystem II; RedCAP: Red lineage chlorophyll a/b-binding-like proteins; ROS: Reactive oxygen species; SEP: Stress-enhanced protein.

## Competing interests

The authors declare that they have no competing interests.

## Authors’ contributions

SS designed and carried out the expression studies. JE designed and carried out the sequence identification and phylogenetic analyses. AG participated in design and realisation of sequence identification, expression and localisation studies. SV carried out the localisation studies. PGK conceived of the study, and participated in its design and coordination. IA conceived of the study, participated in its design and coordination and drafted the manuscript. JL conceived of the study, and participated in its design and coordination. All authors wrote, read and approved the final manuscript.

## Supplementary Material

Additional file 1 **List of sequences, pdf file.** Table S1. List of sequences analysed in Figure 1 and Figure S1 (see Additional file 3). Click here for file

Additional file 2 **Sequence alignment, text file (FASTA format).** Sequence alignment used to build the phylogenetic tree of three- and four-helices protein families of the extended LHC protein superfamily (Figure S3, see Additional file 5), 51 positions, 55 taxa. Click here for file

Additional file 3 **Annotated sequence alignments, pdf file.** Figure S1. (A) Sequence alignment of helices I and III of RedCAPs with red lineage LHCF, CAC/LHCR and LHCX/LI818 proteins. (B) Sequence alignment of helices I and III of RedCAPs with green lineage CAB (LHCa, LHCb and LHCP), LHCSR/LI818, ELIP and LHL4 proteins. (C) Full-length sequence alignment of identified RedCAP amino acid sequences. Identical amino acids are surrounded by black and similar amino acids by grey boxes. Chl-binding motifs located in transmembrane helices I and III are marked with a green bar above the alignment, and the approximate position of transmembrane helix II is marked with a grey bar. Accession numbers of aligned sequences are given in Table S1 (see Additional file 1). Click here for file

Additional file 4 **Putative chlorophyll-binding sites in RedCAPs, pdf file.** Figure S2. Putative chlorophyll-binding sites in members of the LHC (light-harvesting complex) and the RedCAP protein families. Experimentally derived chlorophyll binding sites from *Arabidopsis thaliana* LHCII proteins are indicated in green according to [104-106]. Conserved amino acid positions that may represent putative binding sites for chlorophylls or carotenoids in RedCAPs are indicated in blue. Note that the second helix (helix II) is poorly conserved between distant members of the LHC protein family and not conserved between LHC and RedCAP, therefore the alignment of different helices II does not necessarily show homologous positions. Click here for file

Additional file 5 **Phylogenetic tree, pdf file.** Figure S3. Phylogenetic tree of three- and four-helices protein families of the extended LHC protein superfamily. Robust internal nodes were labelled according to their corresponding statistical support (Maximum likelihood, ML; Neighbor-joining, NJ and bayesian posterior probability). Accession numbers of analysed sequences are listed in Table S1 (see Additional file 1); for the sequence alignment see Additional file 2. Click here for file

Additional file 6 **Sequence alignment, text file (FASTA format).** Sequence alignment used to build the phylogenetic tree of RedCAPs (Figure 1), 146 positions, 13 taxa. Click here for file

Additional file 7 **References for Figure**2**, pdf file.** Table S2. Reference table for the proposed evolutionary history of RedCAP, LHC and LHC-like genes (Figure 2). Findings for cyanobacteria, green algae and plants have been generalised according to published studies, while findings for red algae, cryptophytes and diatoms are specific for representative species of which the plastid-, nucleomorph-, and nuclear genomes (or large transcriptome datasets in the case of “present” statements) have been sequenced and published (see footnotes in table). Genes are marked as “present” if their existence has been reported in the literature; “absent” means that these genes were neither identified in our analyses, nor—to our knowledge—have been reported to exist before (hence no references for “absent” statements). For RedCAP sequence identifiers see also Table 1 of this study. Click here for file

Additional file 8 **Localisation of the RedCAP protein in complex plastids of diatoms II, pdf file.** Figure S4. Expression of the full-length RedCAP:GFP fusion constructs in *P. tricornutum*. Microscopical images of transmitted light (differential interference contrast, DIC), Chlorophyll autofluorescence, GFP fluorescence and a merged image are shown from left to right, fluorescence images are maximum intensity projections of seven slices of a 3.08 *μ*m image stack, scale bars represent 10 *μ*m. Click here for file

Additional file 9 **RedCAP, LHCF and LHC-like gene expression analysis as given by REST, pdf file.** Table S3. RedCAP, LHCF and LHC-like gene expression analysis as given by REST [102]. (A) Data for dark-treated cells (experimental condition D). (B) Data for low light grown cells (experimental condition LL). (C) Data for moderate high light grown cells (experimental condition ML). (D) Data for cells exposed to high light for 2 h (condition HL). Experiments were performed in four replicates as described in the Methods section of the manuscript. For details of the software see [102]; the column “P(H1)” lists the results of REST‘s hypothesis test (the probability that the difference between the sample and control occurs only by chance), the “Result” column lists those up- or down-regulations (relative to the first sample) that are indicated as significant by the statistical randomisation tests by REST. Click here for file

Additional file 10 **Changes in RedCAP, LHC and LHC-like transcript abundance, pdf file.** Figure S5. Changes in RedCAP, LHC and LHC-like transcript abundance. Abbreviations and symbols: upward arrow, up-regulation; rightward arrow, no changes in the expression; downward arrow, down-regulation; CR, diurnal rhythm; HL, high light; LL, low light; ML, moderate high light; n.c., not clear; ^*a*^, a transient, statistically significant up-regulation at the beginning of the previous light phase; ^*b*^, diurnal rhythm under LL but not under ML conditions; ^*c*^, up-regulation in the late phase of illumination; ^*d*^, up-regulation in the early phase of illumination; ^*e*^, short-term moderate up-regulation. Click here for file

Additional file 11 **Primers used for the RT-qPCR, pdf file.** Table S4. Primer sequences used for the real-time quantitative PCR analysis in *P. tricornutum*, the LHCF2 gene has been analysed with the primers designed by Siaut et al. [36] (the gene is called “FcpB” in the cited study). Click here for file
