# Supplementary material for: A novel type of light-harvesting antenna protein of red algal origin in algae with secondary plastids
Source: BMC Evol Biol. 2013 Jul 30;13:159. doi: 10.1186/1471-2148-13-159 (PMC3750529; doi:10.1186/1471-2148-13-159)
Supplement: Additional file 1 — List of sequences, pdf file. Table S1. List of sequences analysed in Figure 1 and Figure S1 (see Additional file 3). [file 1471-2148-13-159-S1.pdf]

Table S1. Table S1. List of sequences analysed in Figure 1 and Figure S1 in additional file 3.

Gene	Organism	Database	Gene model
RedCAP	*Phaeodactylum tricornutum*	JGI	estExt gwp gw1.C chr 10213 [Phatr2:17326]
RedCAP	*Thalassiosira pseudonana*	JGI	JGI CBPB3219.fwd [Thaps3:270215]
RedCAP	*Fragilariopsis cylindrus*	JGI	estExt_Genewise1Plus.C_100147 [Fracy1:210193]
RedCAP	*Guillardia theta*	JGI	f5y_1_estExt_fgenesh2_pm.C_390010 [Guith1:175534]
RedCAP	*Galdieria sulphuraria*	http://genomics.msu.edu/galdieria/	contig 9803
RedCAP	*Griffithsia japonica*	UniProt	Q7XZ09
RedCAP	*Aureococcus anophagefferens*	JGI	e gw1.8.542.1 [Auran1:25646]
RedCAP	*Ectocarpus siliculosus*	Genbank EST	gi242173528
RedCAP	*Emiliania huxleyi*	JGI	estExtDG fgeneshEH pg.C 220091 [Emihu1:463191]; possibly degenerated,too long but with ASAFAP
RedCAP	*Emiliania huxleyi*	JGI	fgenesh_newKGs_pm.28__30 [Emihu1:310333]; used in tree
RedCAP	*Isochrysis galbana*	Genbank EST	gi106825476
RedCAP	*Diacronema lutheri*	Genbank EST	gi106858477
RedCAP	*Gracilaria changii*	Genbank EST	gi120457728
RedCAP	*Pyropia yezoensis*	Genbank EST	gi8590586
RedCAP	*Griffithsia okiensis*	Genbank EST	gi224829379 ;partial EST sequence
RedCAP	*Furcellaria lumbricalis*	Genbank EST	gi294363890 ;partial EST sequence
RedCAP	*Pseudochattonella farcimen*	Genbank EST	gi319967268 ;partial EST sequence
RedCAP	*Gracilaria tenuistipitata*	Genbank EST	gi327362708 ;partial EST sequence
RedCAP	*Porphyridium purpureum*	Genbank EST	gi317790494 ;partial EST sequence
ELIP1	*Arabidopsis thaliana*	UniProt	P93735
ELIP1	*Oryza sativa*	UniProt	Q69W91
ELIP	*Physcomitrella patens*	JGI	estExt fgenesh2 pg.C 780047 [Phypa1 1:233424]
ELIP	*Chlamydomonas reinhardtii*	JGI	estExt fgenesh2 kg.C 170025 [Chlre3:183986]
ELIP (CBR)	*Dunaliella bardawil*	UniProt	P27516
PSBS	*Arabidopsis thaliana*	UniProt	Q9XF91
PSBS	*Oryza sativa*	UniProt	Q7XSS8
PSBS	*Zea mays*	UniProt	Q6WFB1
PSBS	*Physcomitrella patens*	JGI	e gw1.241.24.1 [Phypa1 1:146248]
PSBS	*Chlamydomonas reinhardtii*	JGI	e gwW.1.317.1 [Chlre3:116665]
LHL4	*Chlamydomonas reinhardtii*	JGI	Chlre3:139895
LHL4	*Volvox carteri*	Genbank	D8UBG4
LHL4	*Mesostigma viride*	Genbank	A3QQP2
LHL4	*Micromonas pusilla* strain CCMP1545	Genbank	C1MXA4
LHL4	*Micromonas* sp.RCC299	Genbank EST	gi255085648
LHCr1	*Porphyridium purpureum*	UniProt	AAB39488
LHCr2	*Porphyridium purpureum*	UniProt	AAB39489
CAC2	*Guillardia theta*	Genbank	CAH25343
CAC4	*Guillardia theta*	Genbank	AAF81520
LHCr5	*Galdieria sulphuraria*	Genbank	CAC10534
LHCr4	*Galdieria sulphuraria*	Genbank	CAC87420
LHCf	*Saccharina latissima*	Genbank	AAG13003
LHCf	*Odontella sinensis*	Genbank	Q42395
LHCf	*Ectocarpus siliculosus*	Genbank	CBJ28877
LHCfa	*Phaeodactylum tricornutum*	JGI	estExt_gwp_gw1.C_chr_20047[18049]
LHCfb	*Phaeodactylum tricornutum*	JGI	e gw1.2.411.1 [Phatr2:9669]

Gene	Organism	Database	Gene model
LHCfc	*Phaeodactylum tricornutum*	JGI	e gw1.2.419.1 [Phatr2:10069]
LHCb2.3	*Arabidopsis thaliana*	TAIR; Genbank	At3g27700; NP_189406.1
LHCb1.2	*Arabidopsis thaliana*	TAIR; Genbank	At2g34430; AAG40044.2
LHCb4	*Arabidopsis thaliana*	TAIR; Genbank	AT5g01530; AAK82524.1; CP29
LHCa3	*Arabidopsis thaliana*	TAIR; Genbank	NP_001031217.1; AT1G61520
LHCa1	*Arabidopsis thaliana*	TAIR; Genbank	AT3G54890; BAH20219.1
LHCP	*Mantoniella squamata*	Genbank	AAA20111
LHCx	*Phaeodactylum tricornutum*	Genbank	EEC48746
LHCxa	*Thalassiosira pseudonana*	JGI	e gw1.1.623.1 [Thaps3:31128]
LHCxb	*Thalassiosira pseudonana*	Genbank	XP_002295258
LI818	*Isochrysis galbana*	Genbank	ABA55524
LHCx	*Cychlotella cryptica*	UniProt	CAA04404
LHCSR	*Chlamydomonas reinhardtii*	UniProt	CAA64632
LHCSR	*Volvox carteri*	Genbank	XP_002948670
LI818	*Bigelowiella natans*	Genbank	DAA05894
LHCSR	*Mesostigma viride*	Genbank	DAA05932
LI818	*Physcomitrella patens*	Genbank	XP_001776952
