# Supplementary material for: A novel type of light-harvesting antenna protein of red algal origin in algae with secondary plastids
Source: BMC Evol Biol. 2013 Jul 30;13:159. doi: 10.1186/1471-2148-13-159 (PMC3750529; doi:10.1186/1471-2148-13-159)
Supplement: Additional file 3 — Annotated sequence alignments, pdf file. Figure S1. (A) Sequence alignment of helices I and III of RedCAPs with red lineage LHCF, CAC/LHCR and LHCX/LI818 proteins. (B) Sequence alignment of helices I and III of RedCAPs with green lineage CAB (LHCa, LHCb and LHCP), LHCSR/LI818, ELIP and LHL4 proteins. (C) Full-length sequence alignment of identified RedCAP amino acid sequences. Identical amino acids are surrounded by black and similar amino acids by grey boxes. Chl-binding motifs located in transmembrane helices I and III are marked with a green bar above the alignment, and the approximate position of transmembrane helix II is marked with a grey bar. Accession numbers of aligned sequences are given in Table S1 (see Additional file 1). [file 1471-2148-13-159-S3.pdf]

name	organism	helix I (0 5 10 15 20)	//	helix III (0 5 10 15 20)
RedCAP	Phaeodactylum	QAEKWNGRHAMFGWFFICATAYCKGH		EAEIINGRLAMLGLVMLIGATATSG
RedCAP	Fragilariopsis	HAEKWNGRHAMFGWFFICCTAYAKGH		EAEMINGRLAMLGLISLIFATAIEQ
RedCAP	Thalassiosira	QAEIWNGRHAMFGWVVMCACAYAKGH		EAELANGRMAMMGIITCIAYSGIQG
RedCAP	Aureococcus	KAERWNGRHAMFGWVFIVATGYCQAH		AAEMWNGRLAMLGLVVVSAYSLIYQ
RedCAP	Ectocarpus	KAERWNGRHAMFGWAAIIATGYCQAH		EAEIFNGRMAMMGLVVTATTALTTG
RedCAP	Emiliania	KAERWNGRHAMFGWAAILATGVAKAH		AAEMANGRLAMLGIIAVVITSAISG
RedCAP	Isochrysis	KAERWNGRHAMFGWMAILATGVAKSH		AAEMANGRVAMVGLISVVATSAITG
RedCAP	Diacronema	KAERWNGRHAMFGWVVILATGYAQSH		AAEMMNGRLAMLGLIAAVGASLATG
RedCAP	Galdieria	KAERWNGRHAMFGLLAIVLTGYAKGH		EAELWNGRLAMLGVTFIVATSIITG
RedCAP	Guillardia	NAERWNGRHAMFGIFFMVLSSYLHGR		EAELINGRLAMLGVISITTAALFTG
RedCAP	Gracilaria	KAERWNGRHAMFGFVMLVATAYCKGH		SAELYNCRLAMLGLMVVVGCSVATG
RedCAP	Griffithsia	KAERWNGRHVMFGWLVFWITAYCKGH		DAEIWNGRVAMVGLICLVAQAVGTK
RedCAP	Pyropia	KAERWNGRHVMAGWVILVATGFVKGH		DAEMWNGRVAMLGLMVLVGASVATK
LHCf	Saccharina	YVEVKHGRIAMLAIAGHLTQQNTRLP		AIELNNGRAAQMGILALMVHEELNN
LHCf	Odontella	YVEVKHGRIAHVAFLGQIVTRNGIHL		AIELNNGRAAMMGILGLMVHEQLGG
LHCf	Ectocarpus	YVEIKHGRIAMLAILGHITAQNFRFP		AIELNNGRAAQMGILGLMVHEQINN
LHCf.a	Phaeodactylum	YVEIKHGRICMLAVAGYLTQEAGIRL		AIELNQGRAAQMGILALMVHEQLGV
LHCf.b	Phaeodactylum	YVEIKHGRISMLAVAGYLAQEAGWRL		AIELNQGRAAQMGILALMVHEQLGV
LHCf.c	Phaeodactylum	YVEIKHGRISMLAVVGYLVQEAGVRL		AIELNQGRAAQMGILALMVHEQLGV
LHCr.a1	Porphyridium	EAELKHGRICMLAALGFIYPEIMGGK		LSEVKNGRLAMIAVGGMIHQVLLTK
LHCr.a2	Porphyridium	AAELKHGRIAMLAALGFVVQEILAPK		LSELKNGRLAMIAWTAFAIQQIVTG
CAC2	Guillardia	EAELKHGRIAMLAVLGWLVQESYTFP		LAEIKHGRLAMIAFGGFAHQYFITK
CAC4	Guillardia	EAEIKHGRIAMLAFAGIMVEAAGIKA		LSEIKNGRLAMLAVSGIVHHTLITG
LHCr.5	Galdieria	EAEIKHCRICMLAALGWVFPEFWHLP		TAEIKNGRLAMIAMGAFFHQNLLTN
LHCr.4	Galdieria	ESEIKHGRIAMLAALGFIVQEFVHLP		VAEIKNGRLAMLAVGGFIHHMFLTH
LHCx	Phaeodactylum	EAELTHGRVAMLAVVGFLVGEAVEGS		TKELQNGRLAMLAAAGFMAQELVNG
LHCx.a	Thalassiosira	EAEAQHGRVAMLAAVGMLVTEEPIEY		TKELQNGRLAMLGAAGMIAQELTNG
LHCx.b	Thalassiosira	EAEVTHGRVAMLAVLGFLVGEAVEGS		TKELQNGRLAMLAAAGFLAQEAVDG
Li818	Isochrysis	EAELTHGRVSMLASLGFIIQEKFHPL		EKELSHCRLAMIAASGFLAQEAVSG
LHCx	Cyclotella	EAEVTHGRVAMLAVVGFLVGEAVEGS		TKELQNGRLAMLAAAGFLAQEAVDG

Figure S1 (A)

name	organism	helix I (0 5 10 15 20)	helix III (0 5 10 15 20)
RedCAP	Phaeodactylum	QAEKWNGRHAMFGWFFICATAYCKGH	EAEIINGRLAMLGLVMLIGATATSG
RedCAP	Fragilariopsis	HAEKWNGRHAMFGWFFICCTAYAKGH	EAEMINGRLAMLGLISLIFATAIEQ
RedCAP	Thalassiosira	QAEIWNGRHAMFGWVVMCACAYAKGH	EAELANGRMAMMGIITCIAYSGIQG
RedCAP	Aureococcus	KAERWNGRHAMFGWVFIVATGYCQAH	AAEMWNGRLAMLGLVVVSAYSLIYQ
RedCAP	Ectocarpus	KAERWNGRHAMFGWAAIIATGYCQAH	EAEIFNGRMAMMGLVVTATTALTTG
RedCAP	Emiliania	KAERWNGRHAMFGWAAILATGVAKAH	AAEMANGRLAMLGIIAVVITSAISG
RedCAP	Isochrysis	KAERWNGRHAMFGWMAILATGVAKSH	AAEMANGRVAMVGLISVVATSAITG
RedCAP	Diacronema	KAERWNGRHAMFGWVVILATGYAQSH	AAEMMNGRLAMLGLIAAVGASLATG
RedCAP	Galdieria	KAERWNGRHAMFGLLAIVLTGYAKGH	EAELWNGRLAMLGVTFIVATSIITG
RedCAP	Guillardia	NAERWNGRHAMFGIFFMVLSSYLHGR	EAELINGRLAMLGVISITTAALFTG
RedCAP	Gracilaria	KAERWNGRHAMFGFVMLVATAYCKGH	SAELYNCRLAMLGLMVVVGCSVATG
RedCAP	Griffithsia	KAERWNGRHVMFGWLVFWITAYCKGH	DAEIWNGRVAMVGLICLVAQAVGTK
RedCAP	Pyropia	KAERWNGRHVMAGWVILVATGFVKGH	DAEMWNGRVAMLGLMVLVGASVATK
LHCSR	Chlamydomonas	ESEITHGRVAMLAALGFIVGEQLQDF	TKELNNGRLAMIAIAAFVAQELVEQ
LHCSR	Volvox	ESEIVHGRVSMLAAVGFIVGEQLQDF	TKELNNGRLAMIAIAAFVAQELVEQ
LI818	Bigelowiella	EAEITHSRVAMLAFLGFLFQEALLDR	DKELNNGRLAMLGVAGLMAQELVDG
LHCSR	Mesostigma	ESELVHGRVAMLATLGFVVGETFNPL	TKELNNGRLAMIGIAGMVVQELITQ
LI818	Physcomitrella	ESEITHGRVAMLASLGFIVQEQLQDY	TKELNNGRLAMIAIAAFVAQELVSG
LHCb2.3	Arabidopsis	ELEVIHSRWAMLGALGCTFPEILSKN	VKELKNGRLAMFSMFGFFVQAIVTG
LHCb1.2	Arabidopsis	ELEVIHSRWAMLGALGCVFPELLARN	VKELKNGRLAMFSMFGFFVQAIVTG
LHCb4	Arabidopsis	ECELIHGRWAMLATLGALSVEWLTGV	LAEIKHARLAMVAFLGFAVQAAATG
LHCb5	Arabidopsis	AFELIHARWAMLGAAGFIIPEALNKY	VKEIKNGRLAMFAMLGFFIQAYVTG
LHCa3	Arabidopsis	YGEIINGRFAMLGAAGAIAPEILGKA	LKEVKNGRLAMLAILGYFIQGLVTG
LHCa1	Arabidopsis	ESELIHCRWAMLAVPGILVPEALGYG	VKEIKNGRLALLAFVGFCVQQSAYP
LHCP	Mantoniella	EREVIHGRWAMLGVTGAWAAENGTGI	IKELKHCRLSMFAWLGCIFQALATQ
ELIP	Dunaliella	APEIINGRLAMLGFVAALGAELSTGE	DAEMTNGRFAMIGFAAMLVYEGIQG
ELIP	Chlamydomonas	MPETINGRAAMLGFVAAAGAEIFGSG	ANEKVHGRLAMLGLTTLIIIEMIVG
ELIP	Physcomitrella	APETINGRLAMLGFVWALVGENATGL	KAERWNGRAAMIGFFSLIVTELFLQ
ELIP	Arabidopsis	APERINGRLAMVGFVAALAVELSKGE	DAELWNGRFAMLGLVALAFTEFVKG
ELIP	Oryza	APERINGRLAMVGFVSALAVEASRGG	DAELWNGRFAMLGLVALAFTEFLTG
LHL4	Chlamydomonas	FAETWVGRWSMMGFVSSIVVEFATGK	EVEMTNGRAAMLGFLAAIIVEAGTG
LHL4	Volvox	FSEMWVGRWSMIGFVSSIVVEFATGK	DVELANGRAAMLGFLAAILVEAGTG
LHL4	Mesostigma	FAELWAGRLAMMGFATGAAEELLTGH	NVEMTNGRWAMLGFLAAITVEAATG
LHL4	Micromonas CCMP1545	FAELFIGRTAMGGFATGCIQELLTGD	KVELNNARWAMVGFFAAVLIEARTG
LHL4	Micromonas RCC299	FAELFIGRTAMGGFATGIAQELITGD	SVELNNARWAMVGFAAAVVIEAKTG

Figure S1 (B)

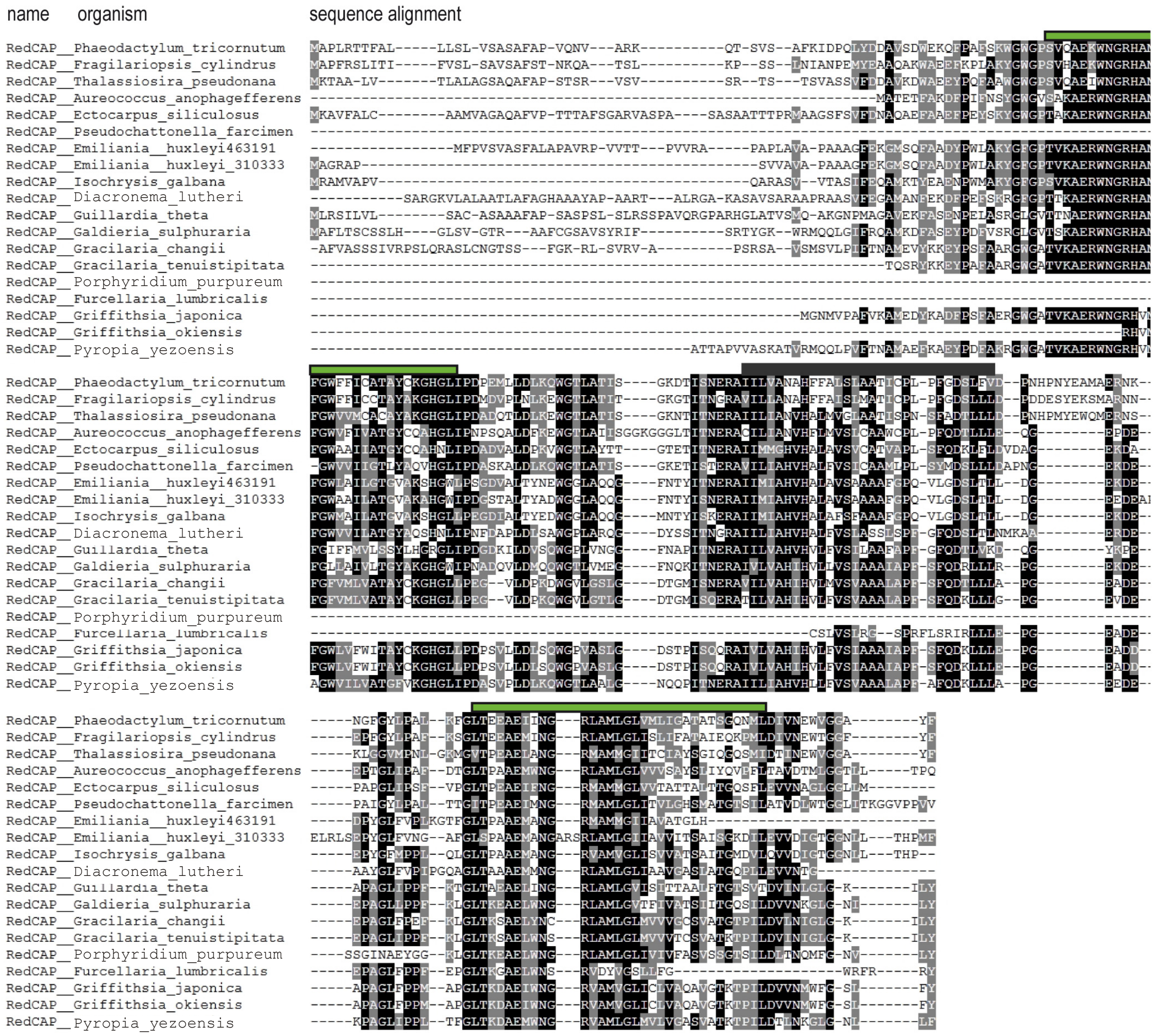
name
organism
sequence alignment
RedCAP__Phaeodactylum_tricornutum
RedCAP__Fragilariopsis_cylindrus
RedCAP__Thalassiosira_pseudonana
RedCAP__Aureococcus_anophagefferens
RedCAP__Ectocarpus_siliculosus
RedCAP__Pseudochattonella_farcimen
RedCAP__Emiliania__huxleyi463191
RedCAP__Emiliania__huxleyi_310333
RedCAP__Isochrysis_galbana
RedCAP__Diacronema_lutheri
RedCAP__Guillardia_theta
RedCAP__Galdieria_sulphuraria
RedCAP__Gracilaria_changii
RedCAP__Gracilaria_tenuistipitata
RedCAP__Porphyridium_purpureum
RedCAP__Furcellaria_lumbricalis
RedCAP__Griffithsia_japonica
RedCAP__Griffithsia_okiensis
RedCAP__Pyropia_yezoensis

Figure S1 (C)
