# Supplementary material for: A novel type of light-harvesting antenna protein of red algal origin in algae with secondary plastids
Source: BMC Evol Biol. 2013 Jul 30;13:159. doi: 10.1186/1471-2148-13-159 (PMC3750529; doi:10.1186/1471-2148-13-159)
Supplement: Additional file 4 — Putative chlorophyll-binding sites in RedCAPs, pdf file. Figure S2. Putative chlorophyll-binding sites in members of the LHC (light-harvesting complex) and the RedCAP protein families. Experimentally derived chlorophyll binding sites from Arabidopsis thaliana LHCII proteins are indicated in green according to [104-106]. Conserved amino acid positions that may represent putative binding sites for chlorophylls or carotenoids in RedCAPs are indicated in blue. Note that the second helix (helix II) is poorly conserved between distant members of the LHC protein family and not conserved between LHC and RedCAP, therefore the alignment of different helices II does not necessarily show homologous positions. [file 1471-2148-13-159-S4.pdf]

name	organism	helix I	helix II	helix III
Lhcbm1	Chlamydomonas	PETFKRYR**E**LELI**H**A**R**WAMLGALGCIFPELLG	ILAILGT**Q**VLLMGAI**E**GY**R**VN	PDTFAELKVK**E**IK**N**G**R**LAMFSMFGFF
Lhcb1.1	Arabidopsis	PETFARNR**E**LEVI**H**S**R**WAMLGALGCVFPELLA	ILAIWAT**Q**VILMGAV**E**GY**R**VA	PEAFAELKVK**E**LK**N**G**R**LAMFSMFGFF
FCP a	Phaeodactylum	QEKFDRLRYVEIKHGRICMLAVAGYLTQEAGI	IIAFIGFLEIAVMKDITGGEF	EDKKLQKRAIELNQGRAAQMGILALM
LHC	Ectocarpus	QERFDRLRYVEIKHGRIAMLAILGHITAQNFR	IFLFIGFLETKVMLQKEGSFP	EEEQATKRAIELNNGRAAQMGILGLM
RedCAP	Phaeodactylum	KWGWGPSVQAEKWNGRHAMFGWFFICATAYCK	IILVA**N**A**H**FFALSLAATICPL	SLFVGLTEEAEIINGRLAMLGLVMLI
RedCAP	Fragilariopsis	XXGWGPSVHAEKWNGRHAMFGWFFICCTAYAK	VILLA**N**A**H**FFAISLMATICPL	SLLLGLTEEAEMINGRLAMLGLISLI
RedCAP	Thalassiosira	AWGWGPSVQAEIWNGRHAMFGWVVMCACAYAK	IILIA**N**V**H**ALMVGLAATISPN	TLLLGVTPEAELANGRMAMMGIITCI
RedCAP	Aureococcus	SYGWGVSAKAERWNGRHAMFGWVFIVATGYCQ	CILIA**N**V**H**FLMVSLCAAWCPL	TLLLGLTPAAEMWNGRLAMLGLVVVS
RedCAP	Ectocarpus	KYGWGPTAKAERWNGRHAMFGWAAIIATGYCQ	IIMMG**H**V**H**ALAVSVCATVAPL	KLFLGLTPEAEIFNGRMAMMGLVVTA
RedCAP	Emiliania	KYGFGPTVKAERWNGRHAMFGWAAILATGVAK	IIMIA**H**V**H**ALAVSAAAAFGPQ	SLTLGLSPAAEMANGRLAMLGIIAVV
RedCAP	Isochrysis	KYGFGPSVKAERWNGRHAMFGWMAILATGVAK	IIMIA**H**V**H**ALAFSFAAAFGPQ	SLTLGLTPAAEMANGRVAMVGLISVV
RedCAP	Diacronema	KRGFGPTTKAERWNGRHAMFGWVVILATGYAQ	IILIA**H**V**H**ALFVSLASSLSPF	SLTLGLTAAAEMMNGRLAMLGLIAAV
RedCAP	Galdieria	SRGLGVTSKAERWNGRHAMFGLLAIVLTGYAK	IVLVA**H**I**H**VLLVSIAAAIAPF	RLLLGLTKEAELWNGRLAMLGVTFIV
RedCAP	Guillardia	SRGLGVTTNAERWNGRHAMFGIFFMVLSSYLH	IILVA**H**V**H**VLFVSILAAFAPF	TLVKGLTAEAELINGRLAMLGVISIT
RedCAP	Gracilaria	ARGWGATVKAERWNGRHAMFGFVMLVATAYCK	VILVA**H**I**H**VLMVSVAAALAPF	TLLLGLTKSAELYNCRLAMLGLMVVV
RedCAP	Griffithsia	ERGWGATVKAERWNGRHVMFGWLVFWITAYCK	IVLVA**H**I**H**VLFVSIAAAIAPF	KLLLGLTKDAEIWNGRVAMVGLICLV
RedCAP	Pyropia	KRGWGATVKAERWNGRHVMAGWVILVATGFVK	IILIA**H**V**H**LLFVSVAAALAPF	KLLLGLTKDAEMWNGRVAMLGLMVLV

Figure S2
