# Supplementary figures and images for: A novel type of light-harvesting antenna protein of red algal origin in algae with secondary plastids
Source: BMC Evol Biol. 2013 Jul 30;13:159. doi: 10.1186/1471-2148-13-159 (PMC3750529; doi:10.1186/1471-2148-13-159)

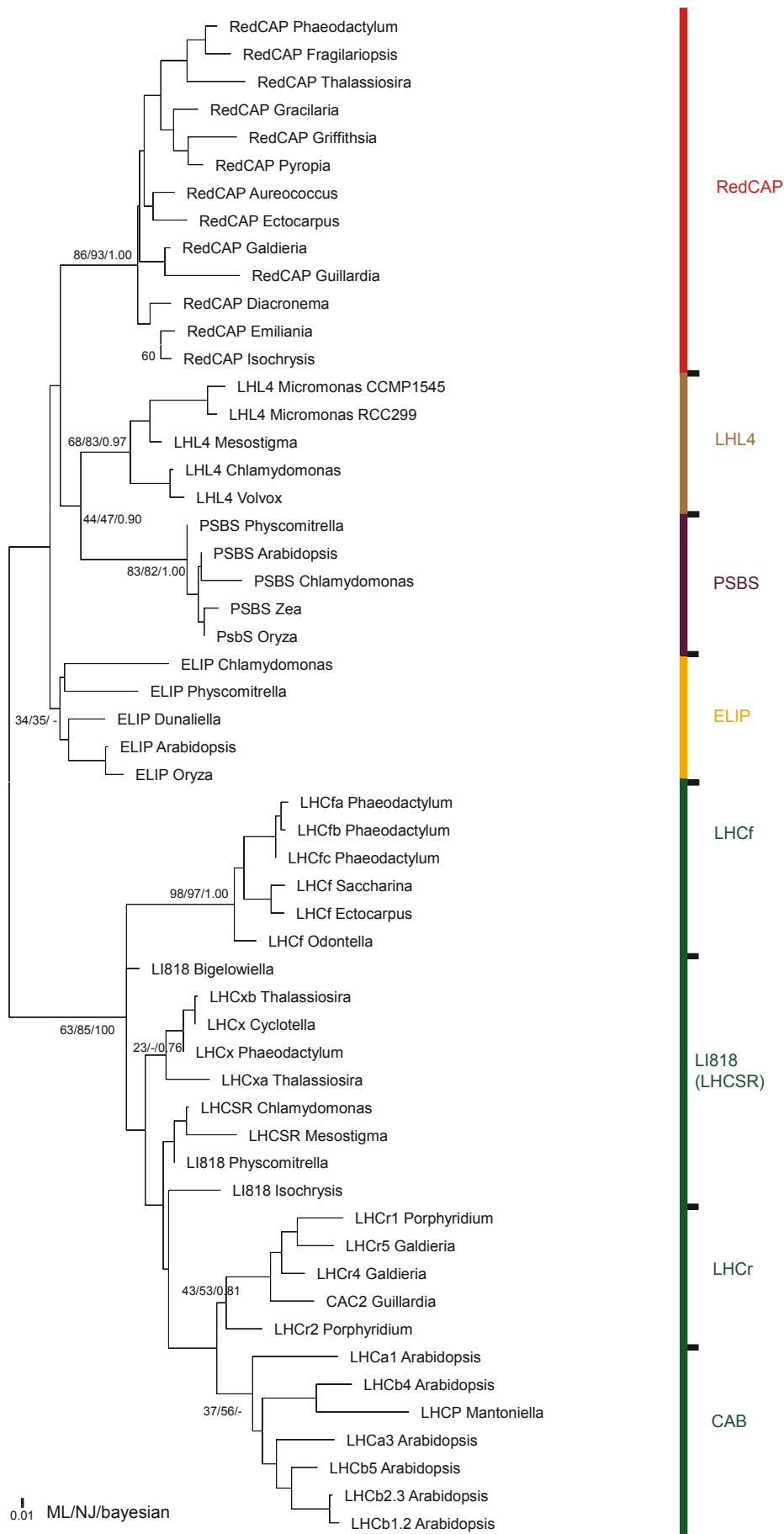

Figure S3

Supplement: Additional file 5 — Phylogenetic tree, pdf file. Figure S3. Phylogenetic tree of three- and four-helices protein families of the extended LHC protein superfamily. Robust internal nodes were labelled according to their corresponding statistical support (Maximum likelihood, ML; Neighbor-joining, NJ and bayesian posterior probability). Accession numbers of analysed sequences are listed in Table S1 (see Additional file 1); for the sequence alignment see Additional file 2. [file 1471-2148-13-159-S5.pdf]

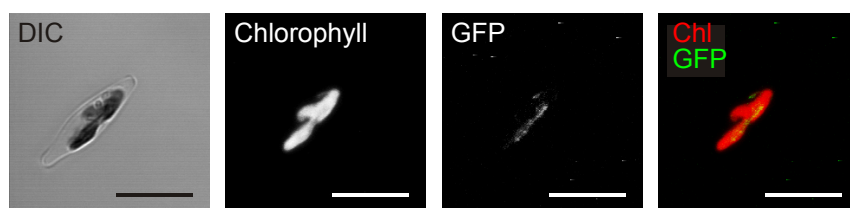

Figure S4

Supplement: Additional file 8 — Localisation of the RedCAP protein in complex plastids of diatoms II, pdf file. Figure S4. Expression of the full-length RedCAP:GFP fusion constructs in P. tricornutum. Microscopical images of transmitted light (differential interference contrast, DIC), Chlorophyll autofluorescence, GFP fluorescence and a merged image are shown from left to right, fluorescence images are maximum intensity projections of seven slices of a 3.08 μm image stack, scale bars represent 10 μm. [file 1471-2148-13-159-S8.pdf]
