# Supplementary material for: A novel type of light-harvesting antenna protein of red algal origin in algae with secondary plastids
Source: BMC Evol Biol. 2013 Jul 30;13:159. doi: 10.1186/1471-2148-13-159 (PMC3750529; doi:10.1186/1471-2148-13-159)
Supplement: Additional file 7 — References for Figure2, pdf file. Table S2. Reference table for the proposed evolutionary history of RedCAP, LHC and LHC-like genes (Figure 2). Findings for cyanobacteria, green algae and plants have been generalised according to published studies, while findings for red algae, cryptophytes and diatoms are specific for representative species of which the plastid-, nucleomorph-, and nuclear genomes (or large transcriptome datasets in the case of “present” statements) have been sequenced and published (see footnotes in table). Genes are marked as “present” if their existence has been reported in the literature; “absent” means that these genes were neither identified in our analyses, nor—to our knowledge—have been reported to exist before (hence no references for “absent” statements). For RedCAP sequence identifiers see also Table 1 of this study. [file 1471-2148-13-159-S7.pdf]

Table S2. Reference table for the proposed evolutionary history of RedCAP, LHC and LHC-like genes (Figure 2). Findings for cyanobacteria, green algae and plants have been generalised according to published studies, while findings for red algae, cryptophytes and diatoms are specific for representative species of which the plastid-, nucleomorph-, and nuclear genomes (or large transcriptome datasets in the case of "present" statements) have been sequenced and published (see footnotes in table). Genes are marked as "present" if their existence has been reported in the literature; "absent" means that these genes were neither identified in our analyses, nor—to our knowledge—have been reported to exist before (hence no references for "absent" statements). For RedCAP sequence identifiers see also Table 1 of this study.

algal group	cyanobacteria	green algae and plants		red algae		cryptophytes			diatoms	
genome	bacterial chromosome	plastid	nuclear	plastid[1]	nuclear[2]	plastid[3]	nucleopmorph[4]	nuclear[5]	plastid[6]	nuclear[7]
HLIP/OHP1	present [1,2]	absent	present [1,2]	present [1–3]	present [1,2]	present [1–3]	absent	absent	absent	present [1,2]
OHP2	absent	absent	present [1,2]	absent	present [1–3]	absent	present[8] [1–3]	absent	absent	present [1–3]
SEP	absent	absent	present [1,2]	absent	present [1]	absent	absent	absent	absent	present [1]
RedCAP	absent	absent	absent	absent	present [1]	absent	absent	present [1]	absent	present [1]
ELIP	absent	absent	present [1,2]	absent	absent	absent	absent	absent	absent	absent
LHC/FCP	absent	absent	present [1,2]	absent	present [1,2]	absent	absent	present [1,2]	absent	present [1,2]

[1] The plastid genomes of *Cyanidioschyzon merolae* [4,5], *Cyanidium caldarium* [5,6], *Porphyra purpurea* [5,7], *Pyropia yezoensis* [5] and *Gracilaria tenuistipitata* var. *liui* [5,8] have been searched.

[2] The *Cyanidioschyzon merolae* Genome Project [9,10] database and the Michigan State University *Galdieria* Database [11–13] have been searched.

[3] The *Guillardia theta* [5,14], *Rhodomonas salina* [5,15] and *Cryptomonas paramecium* [5,16] plastid genomes have been searched.

[4] The *G. theta* [5,17], *C. paramecium* [5,18], *Hemiselmis andersenii* [5,19] and *Chroomonas mesostigmatica* [5,20] nucleomorph genomes have been searched.

[5] The *G. theta* nuclear genome database [21,22] has been searched.

[6] The *Phaeodactylum tricornutum* and *Thalassiosira pseudonana* plastid genomes [5,23] have been searched

[7] The *P. tricornutum* and *T. pseudonana* nuclear genome databases [24–27] have been searched.

[8] OHP2 encoded on chryptophyte nuleomorphs is denoted "Hlip" or "HlipNm" in some studies.

References

[1] Engelken J, Brinkmann H, Adamska I: **Taxonomic distribution and origins of the extended LHC (light-harvesting complex) antenna protein superfamily**. *BMC Evol Biol* 2010, **10**:223.

[2] Green BR: **Evolution of light-harvesting antennas in an oxygen world**. In *Evolution of Primary Producers in the Sea.* Edited by Falkowski PG, Knoll AH, Burlington, USA: Elsevier Academic Press 2007:37–53.

[3] Funk C, Alami M, Tibiletti T, Green BR: **High light stress and the one-helix LHC-like proteins of the cryptophyte *Guillardia theta***. *Biochim Biophys Acta* 2011, **1807**:841–846.

[4] Ohta N, Matsuzaki M, Misumi O, Miyagishima Sy, Nozaki H, Tanaka K, Shin-I T, Kohara Y, Kuroiwa T: **Complete Sequence and Analysis of the Plastid Genome of the Unicellular Red Alga *Cyanidioschyzon merolae***. *DNA Research* 2003, **10**(2):67–77.

[5] **National Center for Biotechnology Information (NCBI)** [http://www.ncbi.nlm.nih.gov].

[6] Glöckner G, Rosenthal A, Valentin K: **The Structure and Gene Repertoire of an Ancient Red Algal Plastid Genome**. *Journal of Molecular Evolution* 2000, **51**:382–390.

[7] Reith M, Munholland J: **Complete nucleotide sequence of the *Porphyra purpurea* chloroplast genome**. *Plant Molecular Biology Reporter* 1995, **13**:333–335.

[8] Hagopian J, Reis M, Kitajima J, Bhattacharya D, Oliveira M: **Comparative Analysis of the Complete Plastid Genome Sequence of the Red Alga *Gracilaria tenuistipitata* var. *liui* Provides Insights into the Evolution of Rhodoplasts and Their Relationship to Other Plastids**. *Journal of Molecular Evolution* 2004, **59**:464–477.

[9] Matsuzaki M, Misumi O, Shin-i T, Maruyama S, Takahara M, Miyagishima Sy, Mori T, Nishida K, Yagisawa F, Nishida K, Yoshida Y, Nishimura Y, Nakao S, Kobayashi T, Momoyama Y, Higashiyama T, Minoda A, Sano M, Nomoto H, Oishi K, Hayashi H, Ohta F, Nishizaka S, Haga S, Miura S, Morishita T, Kabeya Y, Terasawa K, Suzuki Y, Ishii Y, Asakawa S, Takano H, Ohta N, Kuroiwa H, Tanaka K, Shimizu N, Sugano S, Sato N, Nozaki H, Ogasawara N, Kohara Y, Kuroiwa T: **Genome sequence of the ultrasmall unicellular red alga *Cyanidioschyzon merolae* 10D**. *Nature* 2004, **428**(6983):653–657.

[10] **Cyanidioschyzon merolae Genome Project** [http://merolae.biol.s.u-tokyo.ac.jp/].

[11] **Michigan State University Galdieria Database** [http://genomics.msu.edu/galdieria].

[12] Weber A, Oesterhelt C, Gross W, Bräutigam A, Imboden L, Krassovskaya I, Linka N, Truchina J, Schneidereit J, Voll H, Voll L, Zimmermann M, Jamai A, Riekhof W, Yu B, Garavito R, Benning C: **EST-analysis of the thermo-acidophilic red microalga *Galdieriasulphuraria* reveals potential for lipid A biosynthesis and unveils the pathway of carbon export from rhodoplasts**. *Plant Molecular Biology* 2004, **55**:17–32.

[13] Barbier G, Oesterhelt C, Larson MD, Halgren RG, Wilkerson C, Garavito RM, Benning C, Weber AP: **Comparative Genomics of Two Closely Related Unicellular Thermo-Acidophilic Red Algae, *Galdieria sulphuraria* and *Cyanidioschyzon merolae*, Reveals the Molecular Basis of the Metabolic Flexibility of *Galdieria sulphuraria* and Significant Differences in Carbohydrate Metabolism of Both Algae**. *Plant Physiology* 2005, **137**:460–474.

[14] Douglas SE, Penny SL: **The Plastid Genome of the Cryptophyte Alga, *Guillardia theta*: Complete Sequence and Conserved Synteny Groups Confirm Its Common Ancestry with Red Algae**. *J Mol Evol* 1999, **V48**(2):236–244.

[15] Khan H, Parks N, Kozera C, Curtis BA, Parsons BJ, Bowman S, Archibald JM: **Plastid Genome Sequence of the Cryptophyte Alga *Rhodomonas salina* CCMP1319: Lateral Transfer of Putative DNA Replication Machinery and a Test of Chromist Plastid Phylogeny**. *Molecular Biology and Evolution* 2007, **24**(8):1832–1842.

[16] Donaher N, Tanifuji G, Onodera NT, Malfatti SA, Chain PSG, Hara Y, Archibald JM: **The Complete Plastid Genome Sequence of the Secondarily Nonphotosynthetic Alga *Cryptomonas paramecium*: Reduction, Compaction, and Accelerated Evolutionary Rate**. *Genome Biology and Evolution* 2009, **1**:439–448.

[17] Douglas S, Zauner S, Fraunholz M, Beaton M, Penny S, Deng LT, Wu X, Reith M, Cavalier-Smith T, Maier UG: **The highly reduced genome of an enslaved algal nucleus**. *Nature* 2001, **410**:1091–1096.

[18] Tanifuji G, Onodera NT, Wheeler TJ, Dlutek M, Donaher N, Archibald JM: **Complete Nucleomorph Genome Sequence of the Nonphotosynthetic Alga *Cryptomonas paramecium* Reveals a Core Nucleomorph Gene Set**. *Genome Biology and Evolution* 2011, **3**:44–54.

[19] Lane CE, van den Heuvel K, Kozera C, Curtis BA, Parsons BJ, Bowman S, Archibald JM: **Nucleomorph genome of *Hemiselmis andersenii* reveals complete intron loss and compaction as a driver of protein structure and function**. *Proceedings of the National Academy of Sciences* 2007, **104**(50):19908–19913.

[20] Moore CE, Curtis B, Mills T, Tanifuji G, Archibald JM: **Nucleomorph Genome Sequence of the Cryptophyte Alga *Chroomonas mesostigmatica* CCMP1168 Reveals Lineage-Specific Gene Loss and Genome Complexity**. *Genome Biology and Evolution* 2012, **4**(11):1162–1175.

[21] Curtis BA, Tanifuji G, Burki F, Gruber A, Irimia M, Maruyama S, Arias MC, Ball SG, Gile GH, Hirakawa Y, Hopkins JF, Kuo A, Rensing SA, Schmutz J, Symeonidi A, Elias M, Eveleigh RJM, Herman EK, Klute MJ, Nakayama T, Oborník M, Reyes-Prieto A, Armbrust EV, Aves SJ, Beiko RG, Coutinho P, Dacks JB, Durnford DG, Fast NM, Green BR, Grisdale CJ, Hempel F, Henrissat B, Höppner MP, Ishida KI, Kim E, Kořený L, Kroth PG, Liu Y, Malik SB, Maier UG, McRose D, Mock T, Neilson JAD, Onodera NT, Poole AM, Pritham EJ, Richards TA, Rocap G, Roy SW, Sarai C, Schaack S, Shirato S, Slamovits CH, Spencer DF, Suzuki S, Worden AZ, Zauner S, Barry K, Bell C, Bharti AK, Crow JA, Grimwood J, Kramer R, Lindquist E, Lucas S, Salamov A, McFadden GI, Lane CE, Keeling PJ, Gray MW, Grigoriev IV, Archibald JM: **Algal genomes reveal evolutionary mosaicism and the fate of nucleomorphs**. *Nature* 2012, **492**:59–65.

[22] ***Guillardia theta* CCMP2712 v1.0 genome database** [`http://genome.jgi.doe.gov/Guith1/Guith1.home.html`].

[23] Oudot-Le Secq MP, Grimwood J, Shapiro H, Armbrust EV, Bowler C, Green BR: **Chloroplast genomes of the diatoms *Phaeodactylum tricornutum* and *Thalassiosira pseudonana*: comparison with other plastid genomes of the red lineage**. *Mol Gen Genomics* 2007, **277**(4):427–439.

[24] Bowler C, Allen AE, Badger JH, Grimwood J, Jabbari K, Kuo A, Maheswari U, Martens C, Maumus F, Otillar RP, Rayko E, Salamov A, Vandepoele K, Beszteri B, Gruber A, Heijde M, Katinka M, Mock T, Valentin K, Verret F, Berges JA, Brownlee C, Cadoret JP, Chiovitti A, Choi CJ, Coesel S, De Martino A, Detter JC, Durkin C, Falciatore A, Fournet J, Haruta M, Huysman MJJ, Jenkins BD, Jiroutova K, Jorgensen RE, Joubert Y, Kaplan A, Kröger N, Kroth PG, La Roche J, Lindquist E, Lommer M, Martin-Jézéquel V, Lopez PJ, Lucas S, Mangogna M, McGinnis K, Medlin LK, Montsant A, Oudot-Le Secq MP, Napoli C, Obornik M, Parker MS, Petit JL, Porcel BM, Poulsen N, Robison M, Rychlewski L, Rynearson TA, Schmutz J, Shapiro H, Siaut M, Stanley M, Sussman MR, Taylor AR, Vardi A, von Dassow P, Vyverman W, Willis A, Wyrwicz LS, Rokhsar DS, Weissenbach J, Armbrust EV, Green BR, Van de Peer Y, Grigoriev IV: **The *Phaeodactylum* genome reveals the evolutionary history of diatom genomes**. *Nature* 2008, **456**(7219):239–244.

[25] ***Phaeodactylum tricornutum* v2.0 genome database** [`http://genome.jgi-psf.org/Phatr2/Phatr2.home.html`].

[26] Armbrust EV, Berges JA, Bowler C, Green BR, Martinez D, Putnam NH, Zhou S, Allen AE, Apt KE, Bechner M, Brzezinski MA, Chaal BK, Chiovitti A, Davis AK, Demarest MS, Detter JC, Glavina T, Goodstein D, Hadi MZ, Hellsten U, Hildebrand M, Jenkins BD, Jurka J, Kapitonov VV, Kröger N, Lau WWY, Lane TW, Larimer FW, Lippmeier JC, Lucas S, Medina M, Montsant A, Obornik M, Parker MS, Palenik B, Pazour GJ, Richardson PM, Rynearson TA, Saito MA, Schwartz DC, Thamatrakoln K, Valentin K, Vardi A, Wilkerson FP, Rokhsar DS: **The Genome of the Diatom *Thalassiosira pseudonana*: Ecology, Evolution, and Metabolism**. *Science* 2004, **306**(5693):79–86.

[27] ***Thalassiosira pseudonana* v3.0 genome database** [`http://genome.jgi-psf.org/Thaps3/Thaps3.home.html`].
