# Supplementary material for: A novel type of light-harvesting antenna protein of red algal origin in algae with secondary plastids
Source: BMC Evol Biol. 2013 Jul 30;13:159. doi: 10.1186/1471-2148-13-159 (PMC3750529; doi:10.1186/1471-2148-13-159)
Supplement: Additional file 9 — RedCAP, LHCF and LHC-like gene expression analysis as given by REST, pdf file. Table S3. RedCAP, LHCF and LHC-like gene expression analysis as given by REST [102]. (A) Data for dark-treated cells (experimental condition D). (B) Data for low light grown cells (experimental condition LL). (C) Data for moderate high light grown cells (experimental condition ML). (D) Data for cells exposed to high light for 2 h (condition HL). Experiments were performed in four replicates as described in the Methods section of the manuscript. For details of the software see [102]; the column “P(H1)” lists the results of REST‘s hypothesis test (the probability that the difference between the sample and control occurs only by chance), the “Result” column lists those up- or down-regulations (relative to the first sample) that are indicated as significant by the statistical randomisation tests by REST. [file 1471-2148-13-159-S9.pdf]

Table S3. RedCAP, LHCF and LHC-like gene expression analysis as given by REST. (A) Data for dark-treated cells (experimental condition D). (B) Data for low light grown cells (experimental condition LL). (C) Data for moderate high light grown cells (experimental condition ML). (D) Data for cells exposed to high light for two hours (condition HL). Experiments were performed in four replicates as described in the methods section of the manuscript. For details of the software see [1]; the column "P(H1)" lists the results of REST's hypothesis test (the probability that the difference between the sample and control occurs only by chance), the "Result" column lists those up- or down-regulations (relative to the first sample) that are indicated as significant by the statistical randomisation tests by REST.

**A** Dark<br>Time	Gene	Expression	Standard Error	95 % confidence interval	P(H1)	Result
3:00 am	LHCF2	0.187	0.049-1.869	0.016-4.087	0.094	
6:00 am	LHCF2	0.271	0.043-6.207	0.019-13.575	0.342	
9:00 am	LHCF2	0.985	0.269-13.122	0.063-28.698	0.983	
12:00 noon	LHCF2	1.607	0.547-4.581	0.245-6.964	0.483	
3:00 pm	LHCF2	0.219	0.084-0.668	0.029-1.052	0.034	DOWN
6:00 pm	LHCF2	0.121	0.037-0.257	0.024-0.322	0.021	DOWN
9:00 pm	LHCF2	0.075	0.036-0.240	0.009-0.318	0.011	DOWN
12:00 midnight	LHCF2	0.056	0.019-0.136	0.010-0.164	0.005	DOWN
3:00 am	LHCF2	0.054	0.019-0.200	0.006-0.353	0.008	DOWN
6:00 am	LHCF2	0.06	0.022-0.142	0.010-0.191	0.003	DOWN
9:00 am	LHCF2	0.5	0.175-2.451	0.048-4.796	0.399	
3:00 am	OHP1-like1	1.065	0.270-3.408	0.155-6.009	0.89	
6:00 am	OHP1-like1	1.341	0.384-4.673	0.156-10.294	0.721	
9:00 am	OHP1-like1	1.666	0.391-7.758	0.148-17.968	0.565	
12:00 noon	OHP1-like1	0.246	0.054-1.207	0.021-5.542	0.222	
3:00 pm	OHP1-like1	0.252	0.078-1.115	0.020-3.046	0.14	
6:00 pm	OHP1-like1	0.296	0.085-1.444	0.019-4.177	0.171	
9:00 pm	OHP1-like1	0.275	0.087-0.871	0.027-2.393	0.118	
12:00 midnight	OHP1-like1	0.273	0.093-1.020	0.021-3.055	0.164	
3:00 am	OHP1-like1	0.47	0.088-4.036	0.021-11.143	0.482	
6:00 am	OHP1-like1	0.427	0.105-2.398	0.023-6.986	0.41	
9:00 am	OHP1-like1	0.593	0.076-7.831	0.017-20.951	0.712	
3:00 am	OHP1-like2	0.371	0.098-1.432	0.035-4.542	0.294	
6:00 am	OHP1-like2	0.317	0.069-1.468	0.016-5.500	0.294	
9:00 am	OHP1-like2	0.15	0.021-1.028	0.002-3.633	0.168	
12:00 noon	OHP1-like2	0.043	0.008-0.225	0.002-0.859	0.013	DOWN
3:00 pm	OHP1-like2	0.008	0.001-0.059	0.000-0.226	0.015	DOWN
6:00 pm	OHP1-like2	0.011	0.002-0.039	0.001-0.147	0.003	DOWN
9:00 pm	OHP1-like2	0.016	0.004-0.086	0.001-0.274	0.011	DOWN
12:00 midnight	OHP1-like2	0.024	0.005-0.111	0.003-0.429	0.015	DOWN
3:00 am	OHP1-like2	0.029	0.003-0.220	0.002-1.013	0.042	DOWN
6:00 am	OHP1-like2	0.02	0.003-0.100	0.002-0.383	0.005	DOWN
9:00 am	OHP1-like2	0.03	0.004-0.201	0.002-0.773	0.032	DOWN
3:00 am	OHP2	1.34	0.799-2.308	0.579-3.144	0.335	
6:00 am	OHP2	1.761	0.903-3.507	0.711-4.243	0.266	

**A** Dark Time	Gene	Expression	Standard Error	95 % confidence interval	P(H1)	Result
9:00 am	OHP2	2.29	1.741-3.042	1.363-3.718	0.006	UP
12:00 noon	OHP2	1.672	0.785-5.776	0.623-7.867	0.37	
3:00 pm	OHP2	0.583	0.192-1.650	0.139-2.247	0.366	
6:00 pm	OHP2	0.897	0.250-2.914	0.181-3.969	0.846	
9:00 pm	OHP2	0.632	0.259-1.672	0.188-2.277	0.354	
12:00 midnight	OHP2	0.527	0.379-0.696	0.303-0.851	0.02	DOWN
3:00 am	OHP2	0.605	0.256-1.784	0.199-2.430	0.326	
6:00 am	OHP2	0.461	0.172-0.962	0.124-1.176	0.139	
9:00 am	OHP2	1.67	0.983-3.948	0.760-5.378	0.185	
3:00 am	SEPX	0.798	0.534-1.174	0.397-1.685	0.334	
6:00 am	SEPX	0.775	0.487-1.220	0.351-2.045	0.389	
9:00 am	SEPX	0.72	0.468-1.071	0.356-1.624	0.24	
12:00 noon	SEPX	0.328	0.155-0.853	0.110-1.518	0.06	
3:00 pm	SEPX	0.157	0.039-0.578	0.024-0.886	0.032	DOWN
6:00 pm	SEPX	0.185	0.050-0.505	0.030-0.826	0	DOWN
9:00 pm	SEPX	0.148	0.060-0.408	0.037-0.726	0.001	DOWN
12:00 midnight	SEPX	0.223	0.119-0.357	0.080-0.584	0.01	DOWN
3:00 am	SEPX	0.247	0.120-0.506	0.099-0.825	0.016	DOWN
6:00 am	SEPX	0.183	0.108-0.304	0.087-0.464	0.002	DOWN
9:00 am	SEPX	0.364	0.170-0.785	0.135-1.250	0.029	DOWN
3:00 am	RedCAP	0.652	0.204-3.057	0.130-7.900	0.535	
6:00 am	RedCAP	1.294	0.398-8.998	0.201-23.253	0.821	
9:00 am	RedCAP	5.469	2.021-15.661	1.208-38.613	0.01	UP
12:00 noon	RedCAP	3.214	1.529-7.712	0.781-15.019	0.077	
3:00 pm	RedCAP	0.454	0.128-1.406	0.066-2.636	0.299	
6:00 pm	RedCAP	0.296	0.100-0.900	0.056-1.689	0.089	
9:00 pm	RedCAP	0.222	0.082-0.608	0.041-1.278	0.031	DOWN
12:00 midnight	RedCAP	0.205	0.090-0.442	0.054-0.871	0.034	DOWN
3:00 am	RedCAP	0.231	0.089-0.718	0.040-1.302	0.032	DOWN
6:00 am	RedCAP	0.245	0.095-0.672	0.043-1.266	0.058	
9:00 am	RedCAP	1.552	0.462-6.237	0.219-16.118	0.583	

**B** Low light Time	Gene	Expression	Standard Error	95 % confidence interval	P(H1)	Result
3:00 am	LHCF2	0.063	0.028-0.143	0.019-0.192	0.007	DOWN
6:00 am	LHCF2	0.079	0.037-0.193	0.022-0.230	0.019	DOWN
9:00 am	LHCF2	0.114	0.040-0.401	0.030-0.649	0.004	DOWN
12:00 noon	LHCF2	1.418	0.691-2.426	0.438-5.008	0.423	
3:00 pm	LHCF2	5.318	2.813-10.077	1.918-14.203	0	UP
6:00 pm	LHCF2	3.931	2.025-5.598	1.634-10.594	0	UP
9:00 pm	LHCF2	1.004	0.438-2.266	0.298-3.276	0.974	
12:00 midnight	LHCF2	0.041	0.015-0.128	0.013-0.297	0.003	DOWN
3:00 am	LHCF2	0.016	0.007-0.036	0.004-0.083	0.014	DOWN
6:00 am	LHCF2	0.014	0.006-0.032	0.004-0.057	0.011	DOWN
9:00 am	LHCF2	0.054	0.024-0.121	0.017-0.169	0.005	DOWN
3:00 am	OHP1-like1	0.433	0.134-1.368	0.078-2.785	0.287	
6:00 am	OHP1-like1	0.5	0.130-1.735	0.076-3.604	0.326	
9:00 am	OHP1-like1	11.438	3.915-34.612	2.679-54.424	0.044	UP
12:00 noon	OHP1-like1	15.408	4.395-48.403	3.001-113.410	0.005	UP
3:00 pm	OHP1-like1	5.978	1.948-20.388	1.513-25.885	0.006	UP
6:00 pm	OHP1-like1	3.453	1.091-10.034	0.635-20.265	0.101	
9:00 pm	OHP1-like1	5.601	1.745-19.542	0.929-34.577	0.031	UP
12:00 midnight	OHP1-like1	9.962	3.254-31.220	2.003-59.146	0.005	UP
3:00 am	OHP1-like1	2.427	0.695-8.471	0.372-17.169	0.245	
6:00 am	OHP1-like1	2.42	0.900-9.378	0.391-12.448	0.245	
9:00 am	OHP1-like1	45.265	11.778-142.147	8.443-368.972	0.013	UP
3:00 am	OHP1-like2	0.409	0.204-0.815	0.129-1.127	0.056	
6:00 am	OHP1-like2	0.315	0.145-0.660	0.104-0.870	0.001	DOWN
9:00 am	OHP1-like2	0.617	0.297-1.249	0.211-1.591	0.393	
12:00 noon	OHP1-like2	0.792	0.354-2.006	0.184-2.775	0.596	
3:00 pm	OHP1-like2	0.712	0.370-1.509	0.210-2.085	0.451	
6:00 pm	OHP1-like2	0.824	0.372-1.640	0.287-2.119	0.641	
9:00 pm	OHP1-like2	1.302	0.597-2.954	0.354-4.483	0.596	
12:00 midnight	OHP1-like2	1.386	0.688-2.809	0.436-3.871	0.441	
3:00 am	OHP1-like2	0.664	0.281-1.354	0.247-1.583	0.389	
6:00 am	OHP1-like2	0.61	0.275-1.378	0.190-1.667	0.268	
9:00 am	OHP1-like2	0.606	0.260-1.215	0.178-2.149	0.252	
3:00 am	OHP2	0.917	0.351-1.771	0.244-2.280	0.936	
6:00 am	OHP2	1.238	0.585-2.907	0.267-3.842	0.677	
9:00 am	OHP2	3.281	1.282-6.542	0.886-8.020	0.065	
12:00 noon	OHP2	4.601	1.970-10.239	0.947-16.657	0.038	UP
3:00 pm	OHP2	3.067	1.247-6.080	0.763-7.991	0.067	
6:00 pm	OHP2	1.001	0.382-1.725	0.268-3.150	0.983	
9:00 pm	OHP2	0.966	0.389-2.121	0.242-2.512	0.912	
12:00 midnight	OHP2	0.864	0.389-1.869	0.196-2.467	0.869	
3:00 am	OHP2	0.819	0.301-1.562	0.229-1.903	0.772	
6:00 am	OHP2	1.27	0.524-2.550	0.309-3.728	0.636	
9:00 am	OHP2	4.463	1.976-9.945	0.965-14.389	0.049	UP

**B** Low light						
Time	Gene	Expression	Standard Error	95 % confidence interval	P(H1)	Result
3:00 am	SEPX	0.736	0.415-1.108	0.325-1.446	0.338	
6:00 am	SEPX	0.652	0.379-1.104	0.263-1.409	0.202	
9:00 am	SEPX	1.253	0.779-1.986	0.530-2.626	0.434	
12:00 noon	SEPX	1.465	0.757-2.638	0.509-4.023	0.295	
3:00 pm	SEPX	0.876	0.562-1.369	0.339-1.899	0.725	
6:00 pm	SEPX	0.599	0.331-0.949	0.267-1.146	0.096	
9:00 pm	SEPX	0.659	0.419-1.077	0.245-1.495	0.226	
12:00 midnight	SEPX	0.576	0.328-0.873	0.252-1.122	0.08	
3:00 am	SEPX	0.569	0.327-0.884	0.244-1.118	0.077	
6:00 am	SEPX	0.62	0.352-0.992	0.267-1.268	0.133	
9:00 am	SEPX	0.922	0.533-1.475	0.391-1.974	0.735	
3:00 am	RedCAP	0.358	0.056-5.822	0.034-9.752	0.334	
6:00 am	RedCAP	0.687	0.103-11.548	0.068-18.977	0.845	
9:00 am	RedCAP	2.53	0.403-38.300	0.231-75.020	0.614	
12:00 noon	RedCAP	11.094	1.590-195.756	0.752-376.378	0.038	UP
3:00 pm	RedCAP	18.317	2.550-321.600	1.732-524.814	0.002	UP
6:00 pm	RedCAP	8.538	1.208-166.812	0.891-203.386	0.027	UP
9:00 pm	RedCAP	2.411	0.361-45.612	0.194-69.050	0.589	
12:00 midnight	RedCAP	0.14	0.018-1.579	0.006-7.262	0.159	
3:00 am	RedCAP	0.189	0.028-3.285	0.015-5.617	0.209	
6:00 am	RedCAP	0.189	0.028-3.414	0.018-5.106	0.197	
9:00 am	RedCAP	0.624	0.088-8.525	0.044-22.767	0.736	

**C** Moderate high light Time	Gene	Expression	Standard Error	95 % confidence interval	P(H1)	Result
3:00 am	LHCF2	0.345	0.058-4.312	0.008-14.486	0.429	
6:00 am	LHCF2	0.492	0.092-8.360	0.005-26.378	0.545	
9:00 am	LHCF2	0.012	0.003-0.149	0.003-0.182	0.057	
12:00 noon	LHCF2	0.004	0.001-0.040	0.001-0.067	0.029	DOWN
3:00 pm	LHCF2	0.023	0.006-0.204	0.004-0.484	0.056	
6:00 pm	LHCF2	0.082	0.022-0.955	0.017-1.409	0.113	
9:00 pm	LHCF2	0.09	0.024-1.027	0.017-1.634	0.111	
12:00 midnight	LHCF2	0.081	0.024-0.863	0.016-1.426	0.082	
3:00 am	LHCF2	0.162	0.049-1.582	0.029-3.088	0.155	
6:00 am	LHCF2	0.17	0.050-1.843	0.033-3.003	0.138	
9:00 am	LHCF2	0.015	0.004-0.148	0.003-0.283	0.016	DOWN
3:00 am	OHP1-like1	0.425	0.064-2.319	0.041-5.599	0.412	
6:00 am	OHP1-like1	0.584	0.096-4.602	0.045-5.764	0.572	
9:00 am	OHP1-like1	3.852	0.203-80.016	0.144-326.574	0.378	
12:00 noon	OHP1-like1	26.983	3.931-205.333	2.477-267.220	0.012	UP
3:00 pm	OHP1-like1	16.216	3.245-159.529	0.750-211.650	0.124	
6:00 pm	OHP1-like1	14.708	2.015-120.589	1.271-151.140	0.01	UP
9:00 pm	OHP1-like1	6.829	0.682-76.213	0.407-103.528	0.155	
12:00 midnight	OHP1-like1	10.136	1.055-107.051	0.665-133.960	0.127	
3:00 am	OHP1-like1	1.922	0.301-9.639	0.240-17.560	0.484	
6:00 am	OHP1-like1	1.356	0.207-7.068	0.158-13.088	0.734	
9:00 am	OHP1-like1	13.224	1.698-154.522	0.585-200.610	0.069	
3:00 am	OHP1-like2	0.406	0.201-0.904	0.145-1.514	0.089	
6:00 am	OHP1-like2	0.232	0.060-0.684	0.038-0.943	0.02	DOWN
9:00 am	OHP1-like2	0.265	0.114-0.527	0.089-0.826	0.013	DOWN
12:00 noon	OHP1-like2	0.804	0.318-2.080	0.218-2.890	0.688	
3:00 pm	OHP1-like2	1.442	0.924-2.220	0.718-3.112	0.275	
6:00 pm	OHP1-like2	2.227	1.179-4.226	0.897-5.839	0.06	
9:00 pm	OHP1-like2	2.228	1.091-4.495	0.793-6.200	0.085	
12:00 midnight	OHP1-like2	2.508	1.908-3.753	1.313-4.468	0.002	UP
3:00 am	OHP1-like2	1.538	0.916-2.569	0.585-3.497	0.211	
6:00 am	OHP1-like2	1.043	0.714-1.505	0.528-2.076	0.734	
9:00 am	OHP1-like2	0.558	0.336-0.923	0.241-1.518	0.1	
3:00 am	OHP2	1.785	1.405-2.298	1.262-2.939	0.029	UP
6:00 am	OHP2	1.217	0.344-2.460	0.269-3.183	0.718	
9:00 am	OHP2	1.663	1.418-2.006	1.286-2.208	0.023	UP
12:00 noon	OHP2	2.671	1.258-3.923	0.983-4.891	0.06	
3:00 pm	OHP2	2.714	1.770-5.201	1.553-6.367	0.03	UP
6:00 pm	OHP2	2.738	2.071-3.492	1.654-4.105	0.025	UP
9:00 pm	OHP2	2.408	1.685-3.188	1.392-3.943	0.025	UP
12:00 midnight	OHP2	2.637	1.921-3.650	1.641-4.549	0.029	UP
3:00 am	OHP2	1.887	1.245-2.615	0.973-3.053	0.056	
6:00 am	OHP2	1.688	1.295-2.187	1.012-2.473	0.045	UP
9:00 am	OHP2	1.914	1.363-2.689	1.170-3.242	0.031	UP

**C** Moderate high light						
Time	Gene	Expression	Standard Error	95 % confidence interval	P(H1)	Result
3:00 am	SEPX	1.06	0.868-1.303	0.834-1.598	0.645	
6:00 am	SEPX	0.596	0.417-0.802	0.361-1.069	0.052	
9:00 am	SEPX	0.945	0.748-1.161	0.735-1.382	0.598	
12:00 noon	SEPX	1.194	0.502-1.900	0.416-2.257	0.721	
3:00 pm	SEPX	1.042	0.725-1.522	0.711-2.069	0.823	
6:00 pm	SEPX	0.83	0.667-1.036	0.604-1.261	0.247	
9:00 pm	SEPX	0.684	0.509-0.798	0.478-1.121	0.077	
12:00 midnight	SEPX	0.757	0.514-0.956	0.428-1.349	0.18	
3:00 am	SEPX	0.768	0.601-0.894	0.586-1.208	0.079	
6:00 am	SEPX	0.778	0.608-0.979	0.531-1.201	0.103	
9:00 am	SEPX	0.606	0.408-1.249	0.392-1.649	0.138	
3:00 am	RedCAP	0.512	0.152-3.696	0.043-7.709	0.441	
6:00 am	RedCAP	0.941	0.308-8.666	0.045-16.500	0.801	
9:00 am	RedCAP	0.025	0.010-0.170	0.009-0.227	0.045	DOWN
12:00 noon	RedCAP	0.019	0.006-0.119	0.002-0.284	0.019	DOWN
3:00 pm	RedCAP	0.06	0.021-0.333	0.014-0.661	0.04	DOWN
6:00 pm	RedCAP	0.107	0.037-0.616	0.032-1.132	0.071	
9:00 pm	RedCAP	0.106	0.036-0.600	0.023-1.294	0.071	
12:00 midnight	RedCAP	0.089	0.037-0.637	0.033-0.758	0.007	DOWN
3:00 am	RedCAP	0.32	0.126-2.263	0.070-3.297	0.138	
6:00 am	RedCAP	0.492	0.195-3.215	0.122-5.171	0.365	
9:00 am	RedCAP	0.02	0.008-0.145	0.007-0.183	0.008	DOWN

**D**											
**Low light**	(control)						**High light**	(treatment)			
Time	Gene	Expression	Standard Error	95 % confidence interval	P(H1)	Result	Expression	Standard Error	95 % confidence interval	P(H1)	Result
15 min	LHCF2	6.28	1.721-22.891	1.708-23.070	0	UP	0.23	0.180-0.298	0.170-0.315	0	DOWN
30 min	LHCF2	6.69	1.835-24.395	1.825-24.531	0	UP	0.01	0.007-0.009	0.007-0.010	0	DOWN
45 min	LHCF2	7.71	2.114-28.134	2.087-28.494	0	UP	0.01	0.003-0.008	0.002-0.008	0	DOWN
60 min	LHCF2	7.77	2.129-28.400	2.061-29.316	0	UP	0.00	0.002-0.003	0.002-0.003	0	DOWN
120 min	LHCF2	5.87	1.615-21.726	1.435-24.111	0	UP	0.00	0.002-0.003	0.002-0.003	0	DOWN
recov 4 h	LHCF2	3.27	0.896-11.968	0.852-12.542	0.65		0.02	0.003-0.078	0.003-0.082	0	DOWN
15 min	OHP1-like1	1.20	0.994-1.459	0.986-1.471	0.507		30.71	25.050-38.257	22.510-42.091	0.337	
30 min	OHP1-like1	1.40	1.347-1.451	1.336-1.463	0.167		23.52	18.707-30.030	16.810-33.039	0.164	
45 min	OHP1-like1	1.25	1.031-1.523	1.022-1.535	0	UP	11.64	8.706-15.818	7.823-17.403	0	UP
60 min	OHP1-like1	1.36	1.082-1.706	1.073-1.721	0	UP	3.52	3.240-3.846	3.121-3.965	0	UP
120 min	OHP1-like1	0.87	0.847-0.896	0.840-0.904	0	DOWN	2.36	1.986-2.849	1.785-3.134	0	UP
recov 4 h	OHP1-like1	0.52	0.390-0.691	0.387-0.697	0.177		0.23	0.121-0.458	0.108-0.504	0	DOWN
15 min	OHP1-like2	0.82	0.724-0.931	0.674-0.995	0.334		0.01	0.010-0.012	0.010-0.013	0	DOWN
30 min	OHP1-like2	0.94	0.832-1.068	0.778-1.137	0.337		0.01	0.005-0.006	0.005-0.006	0.158	
45 min	OHP1-like2	0.91	0.803-1.028	0.772-1.066	0.328		0.01	0.004-0.009	0.004-0.009	0	DOWN
60 min	OHP1-like2	0.96	0.850-1.090	0.802-1.151	1		0.00	0.004-0.005	0.004-0.005	0	DOWN
120 min	OHP1-like2	0.87	0.773-0.990	0.738-1.035	0.331		0.01	0.004-0.009	0.004-0.010	0	DOWN
recov 4 h	OHP1-like2	1.58	1.395-1.778	1.393-1.780	0	UP2	0.61	0.143-2.636	0.133-2.813	0.673	
15 min	OHP2	0.80	0.586-1.090	0.545-1.165	0.494		0.81	0.700-0.942	0.649-1.011	0.33	
30 min	OHP2	0.81	0.742-0.888	0.696-0.943	0	DOWN	0.10	0.079-0.130	0.070-0.145	0	DOWN
45 min	OHP2	0.62	0.516-0.744	0.480-0.795	0	DOWN	0.04	0.031-0.051	0.027-0.057	0	DOWN
60 min	OHP2	0.67	0.504-0.894	0.469-0.956	0.155		0.02	0.015-0.018	0.015-0.019	0	DOWN
120 min	OHP2	0.41	0.352-0.482	0.328-0.516	0	DOWN	0.02	0.014-0.035	0.012-0.039	0.341	
recov 4 h	OHP2	0.20	0.187-0.224	0.177-0.236	0	DOWN	0.19	0.111-0.329	0.098-0.366	0	DOWN
15 min	SEPX	0.90	0.789-1.036	0.722-1.124	0.676		2.76	2.143-3.610	1.915-3.990	0	UP
30 min	SEPX	0.88	0.774-1.009	0.745-1.047	0.337		1.15	0.869-1.609	0.693-1.928	0.494	
45 min	SEPX	0.75	0.658-0.858	0.636-0.886	0	DOWN	0.50	0.390-0.651	0.382-0.665	0.174	
60 min	SEPX	0.74	0.648-0.848	0.612-0.895	0.155		0.13	0.112-0.158	0.103-0.167	0	DOWN
120 min	SEPX	0.52	0.458-0.600	0.423-0.645	0	DOWN	0.07	0.055-0.091	0.053-0.093	0.178	
recov 4 h	SEPX	0.41	0.362-0.474	0.340-0.502	0	DOWN	0.58	0.243-1.477	0.194-1.770	0.673	
15 min	RedCAP	1.07	0.686-1.664	0.640-1.774	0.658		0.08	0.071-0.089	0.069-0.091	0	DOWN
30 min	RedCAP	1.25	0.833-2.062	0.633-2.543	0.504		0.01	0.010-0.013	0.009-0.014	0	DOWN
45 min	RedCAP	1.00	0.642-1.560	0.588-1.690	0.663		0.01	0.009-0.019	0.008-0.021	0.344	
60 min	RedCAP	1.00	0.641-1.558	0.592-1.676	1		0.01	0.008-0.010	0.008-0.010	0	DOWN
120 min	RedCAP	0.64	0.414-1.014	0.349-1.169	0.346		0.01	0.009-0.013	0.008-0.014	0	DOWN
recov 4 h	RedCAP	0.16	0.104-0.252	0.095-0.275	0	DOWN	0.02	0.010-0.039	0.009-0.043	0	DOWN

References

[1] Pfaffl MW, Horgan GW, Dempfle L: **Relative expression software tool (REST) for group-wise comparison and statistical analysis of relative expression results in real-time PCR**. *Nucleic Acids Res* 2002, **30**:e36.
