# Supplementary material for: A novel type of light-harvesting antenna protein of red algal origin in algae with secondary plastids
Source: BMC Evol Biol. 2013 Jul 30;13:159. doi: 10.1186/1471-2148-13-159 (PMC3750529; doi:10.1186/1471-2148-13-159)
Supplement: Additional file 10 — Changes in RedCAP, LHC and LHC-like transcript abundance, pdf file. Figure S5. Changes in RedCAP, LHC and LHC-like transcript abundance. Abbreviations and symbols: upward arrow, up-regulation; rightward arrow, no changes in the expression; downward arrow, down-regulation; CR, diurnal rhythm; HL, high light; LL, low light; ML, moderate high light; n.c., not clear; a, a transient, statistically significant up-regulation at the beginning of the previous light phase; b, diurnal rhythm under LL but not under ML conditions; c, up-regulation in the late phase of illumination; d, up-regulation in the early phase of illumination; e, short-term moderate up-regulation. [file 1471-2148-13-159-S10.pdf]

Gene	D	LL	ML	HL	CR
RedCAP	↓[a]	↑	↓	↓	yes[b]
LHCf	↓	↑	↓	↓	yes[b]
OHP1-like 1	↓	↑	↑	↑	n.c.
OHP1-like 2	↓	→	↑[c]	↓	no
OHP2	↓[a]	↑[d]	↑	↓	yes
SEPx	↓	→	→	↑[e]	no

Figure S5
