# Supplementary material for: A novel type of light-harvesting antenna protein of red algal origin in algae with secondary plastids
Source: BMC Evol Biol. 2013 Jul 30;13:159. doi: 10.1186/1471-2148-13-159 (PMC3750529; doi:10.1186/1471-2148-13-159)
Supplement: Additional file 11 — Primers used for the RT-qPCR, pdf file. Table S4. Primer sequences used for the real-time quantitative PCR analysis in P. tricornutum, the LHCF2 gene has been analysed with the primers designed by Siaut et al. [36] (the gene is called “FcpB” in the cited study). [file 1471-2148-13-159-S11.pdf]

Table S4. Primer sequences used for the real-time quantitative PCR analysis in *P. tricornutum*, the LHCF2 gene has been analysed with the primers designed by Siaut et al. [36] (the gene is called "FcpB" in the cited study).

Primer name	Primer sequence 5´-3´
RedCAP For	CTTTGTCGACCCTAACCACCCT
RedCAP Rev	TTCGGCTTCTTCGGTAAGTCC
LHCF2 For	GCCGATATCCCCAATGGATTT
LHCF2 Rev	CTTGGTCGAAGGAGTCCCATC
OHP1-like 1 For	GCCATCGGAAACCGAGAAA
OHP1-like 1 Rev	CGAGTTCGACCGTATCCAATG
OHP1-like 2 For	GCGCCAACCACTCTTTTCTG
OHP1-like 2 Rev	GGGTAATACCAGTAACTGTGCCAAA
OHP2 For	AAGAAACCTGGCGGAAGGAA
OHP2 Rev	AGGAAGAACATGGCGAATCGT
SEPX For	GCAGAAATTTGGAATGGACGTG
SEPX Rev	CGCCTTCCTGTAGACCCTGAAT
18S rRNA For	TGCCCTTTGTACACACCGC
18 rRNA Rev	AAGTTCTCGCAACCAACACCA

Abbreviations: For, forward primer, Rev, reverse primer.
